# Perfect prosthetic heart valve: generative design with machine learning, modeling, and optimization

**DOI:** 10.3389/fbioe.2023.1238130

**Published:** 2023-09-15

**Authors:** Viacheslav V. Danilov, Kirill Y. Klyshnikov, Pavel S. Onishenko, Alex Proutski, Yuriy Gankin, Farid Melgani, Evgeny A. Ovcharenko

**Affiliations:** ^1^ Politecnico di Milano, Milan, Italy; ^2^ Quantori, Cambridge, MA, United States; ^3^ Research Institute for Complex Issues of Cardiovascular Diseases, Kemerovo, Russia; ^4^ University of Trento, Trento, Italy

**Keywords:** generative design, heart valve prosthesis, prosthetic heart valve, machine learning, optimization, gradient methods, computer-aided design, finite element method

## Abstract

Majority of modern techniques for creating and optimizing the geometry of medical devices are based on a combination of computer-aided designs and the utility of the finite element method This approach, however, is limited by the number of geometries that can be investigated and by the time required for design optimization. To address this issue, we propose a generative design approach that combines machine learning (ML) methods and optimization algorithms. We evaluate eight different machine learning methods, including decision tree-based and boosting algorithms, neural networks, and ensembles. For optimal design, we investigate six state-of-the-art optimization algorithms, including Random Search, Tree-structured Parzen Estimator, CMA-ES-based algorithm, Nondominated Sorting Genetic Algorithm, Multiobjective Tree-structured Parzen Estimator, and Quasi-Monte Carlo Algorithm. In our study, we apply the proposed approach to study the generative design of a prosthetic heart valve (PHV). The design constraints of the prosthetic heart valve, including spatial requirements, materials, and manufacturing methods, are used as inputs, and the proposed approach produces a final design and a corresponding score to determine if the design is effective. Extensive testing leads to the conclusion that utilizing a combination of ensemble methods in conjunction with a Tree-structured Parzen Estimator or a Nondominated Sorting Genetic Algorithm is the most effective method in generating new designs with a relatively low error rate. Specifically, the Mean Absolute Percentage Error was found to be 11.8% and 10.2% for lumen and peak stress prediction respectively. Furthermore, it was observed that both optimization techniques result in design scores of approximately 95%. From both a scientific and applied perspective, this approach aims to select the most efficient geometry with given input parameters, which can then be prototyped and used for subsequent *in vitro* experiments. By proposing this approach, we believe it will replace or complement CAD-FEM-based modeling, thereby accelerating the design process and finding better designs within given constraints. The repository, which contains the essential components of the study, including curated source code, dataset, and trained models, is publicly available at https://github.com/ViacheslavDanilov/generative_design.

## Introduction

The pathological changes in heart valves, including stenosis or regurgitation, have emerged as a major focus of contemporary cardiovascular medicine (Coffey et al., 2021). Valvular Heart Disease (VHD), resulting from such pathological changes, is a highly prevalent problem, affecting 75.2 million individuals globally and causing over 0.5 million deaths annually (Roth et al., 2020). Current clinical recommendations for treatment of VHD primarily focus on pharmacological therapy as a symptomatic correction, while acknowledging that it cannot influence the underlying valvular pathology. Thus, surgical or transcatheter replacement of heart valves with artificial products is considered the main treatment strategy (Vahanian et al., 2022).

Prosthetic heart valves (PHVs), made from biologically derived materials that mimic the anatomical structure of the tricuspid valve, are the premier option used to replace malfunctioning heart valves. Although bioprostheses exhibit superior hemodynamics compared to mechanical alternatives, studies have revealed their susceptibility to structural failure within 10–15 years (Côté et al., 2017; Bourguignon et al., 2018). This failure is a multivariate process involving mechanical degradation, material stress accumulation, and the active participation of proteolytic enzymes and blood cells, leading to the deposition of calcium ions on the leaflet material (Kostyunin et al., 2020). The observed relationship between stress distribution characteristics of the valves and their duration of operation indicates the need to optimize the valve design in terms of shape and geometry (Vesely, 2003; Abbasi et al., 2019).

This optimization problem is relevant for most types of prosthetic heart valves, including surgically framed, transcatheter, and the emerging field of polymer prostheses, as they use similar materials and operating principles. Achieving a generalized optimization algorithm is hindered by a multitude of factors. For example, PHVs require several parameters to describe their leaflets, depending on their level of complexity. Furthermore, leaflets may be made of materials with pronounced nonlinear properties, such as anisotropy and fiber orientation. In addition, biomechanical modeling of certain PHV models may require the inclusion of surrounding elements, such as a support frame or recipient tissues, in the analysis. Despite these difficulties, various research groups have proposed techniques for optimizing different types of PHVs, primarily based on numerical methods implemented through computer-aided engineering software, usually focusing on the finite element method. Resulting in three general approaches to the optimization of the PHV valve apparatus:

1. Manual approach. This approach involves independent modification of the valve geometry based on the designer’s subjective experience, with the effectiveness being assessed through *in silico* simulation (Li and Sun, 2010; Xu et al., 2018)*.* While this method can be useful in the early stages of prosthesis development, its results may be limited due to the subjective nature of the optimization and the lack of systematic consideration of the entire space of possible shapes.
2. Semi-automatic approach. To address the subjectivity of manual optimization, researchers have proposed a semi-automatic approach that partially automates the valve design process. This involves generating a variety of three-dimensional valve models, performing finite element analysis to study the stress-strain state, and selecting the optimal shape (Hsu et al., 2015; Li and Sun, 2017; Abbasi and Azadani, 2020). However, the finite element method, especially in the context of modeling the effect of blood flow (Fluid-Structure Interaction, FSI), can become computationally intensive, potentially limiting a comprehensive investigation of the entire spectrum of feasible geometric parameters.
3. ML approach. To overcome the limitations of FEM, some authors have proposed to replace it with a ML based surrogate method (Nallagonda, 2018; Balu et al., 2019; Liang and Sun, 2019; Gulbulak et al., 2021). This involves imitating the numerical modeling process with ML algorithms to quickly obtain results. This approach can significantly increase the productivity of PHV research, increasing the number of geometries under investigation by an order of magnitude (Nallagonda, 2018; Balu et al., 2019; Liang and Sun, 2019). However, the number of combinations grows exponentially as new properties are introduced or the range of their values is expanded, making this approach challenging to apply in practice.

To address the limitations of existing approaches, this study focuses on the use of iterative algorithms for optimal geometry determination. Specifically, we demonstrate the effectiveness of combining ML and optimization algorithms. ML algorithms, trained on a sample of 11,565 designs, accurately evaluate key geometric characteristics of PHV valves whereas, optimization algorithms quantify the selected leaflet geometry through the utility of a custom function. The geometry is then iteratively modified and re-evaluated until an optimal design is obtained, within the specified constraints. This approach to optimization is not based on a comprehensive exploration of all possible parameter combinations, but rather on the iterative modification of the leaflet geometry to bring its characteristics to an optimal state.

**FIGURE 1:**
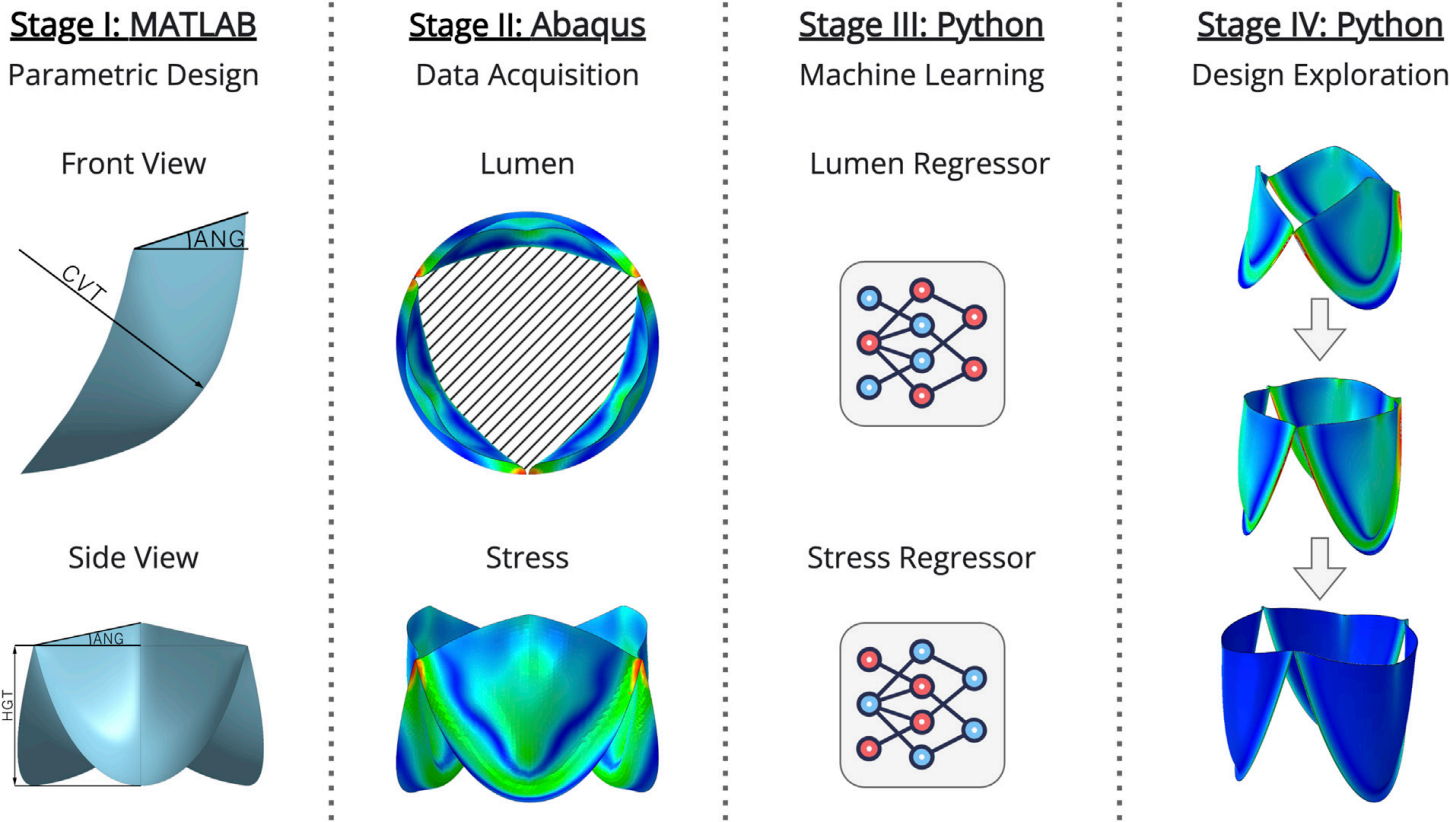
Overview of the proposed multistage generative approach.

Here, the key concept is the ability to evaluate critical parameters of a prototype device, without the need for additional experiments, using FEM. The proposed method, as shown in Figure 1, combines ML and optimization algorithms to replace FEM-based modeling, thereby speeding up the search for optimal geometry parameters of the generated PHVs. From both a scientific and an applied perspective, this approach aims to select the most efficient PHV geometry with specific input parameters, which can then be prototyped and tested in subsequent *in vitro* experiments.

The proposed research consists of:

• Implementing a parametric valve design (detailed in the “Parametric Design of Valves” section).
• Acquiring initial PHV designs using FEM (detailed in the “Data Collection” section).
• Training and validating ML models using the AutoML methodology (detailed in the “Application of Machine Learning in Generative Design” section).
• Searching for optimal designs using an optimization algorithm (detailed in the “Exploration of Optimal Designs” section).
• Testing the generated PHV designs using finite element modeling (detailed in the “Finite Element Analysis of Generated PHV Designs” section).

## Methods

### Parametric design of valves

**FIGURE 2:**
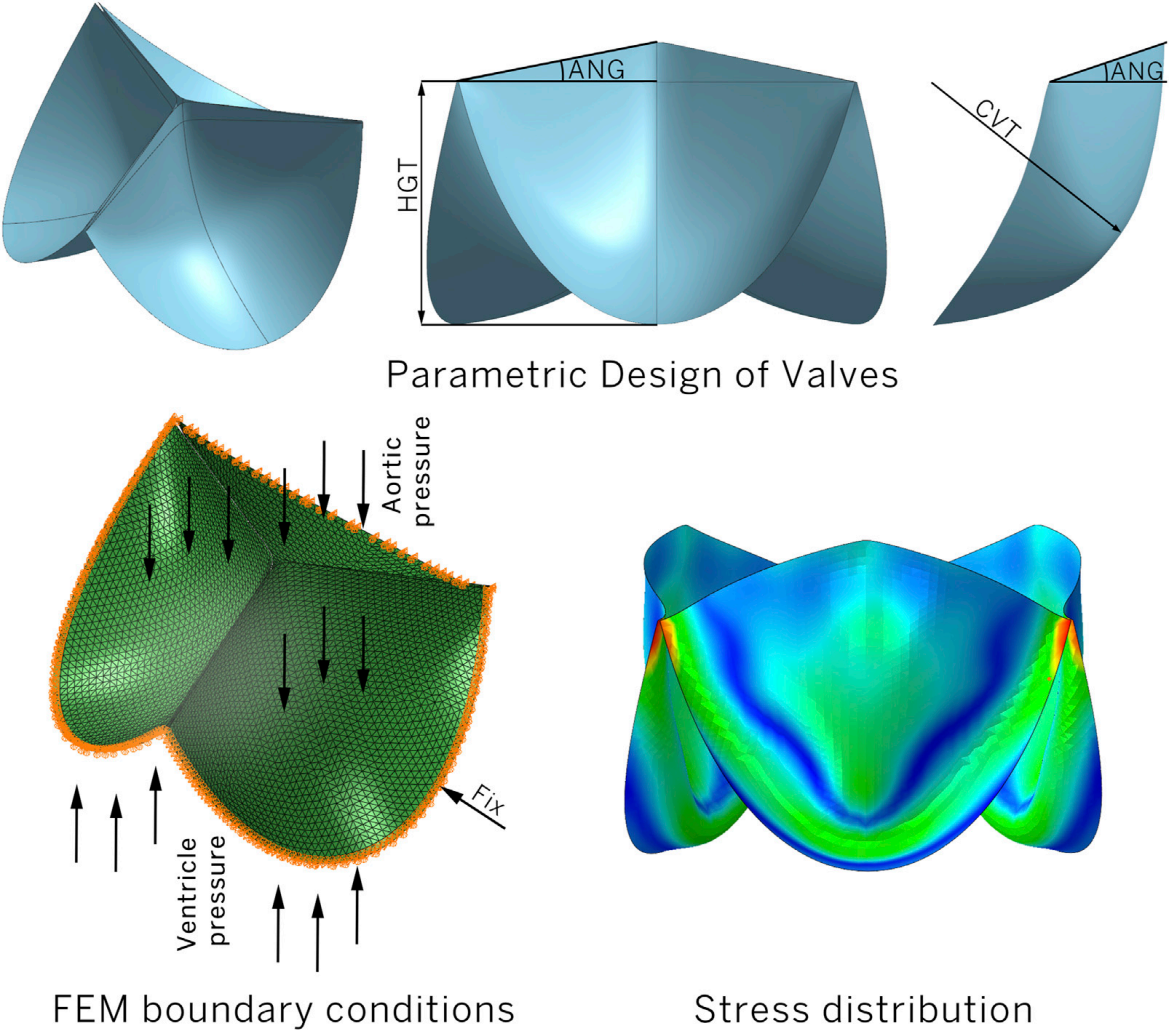
3D model of a PHV with parametric variations.

The study is based on a three-dimensional parametric model of a leaflet prosthesis for the mitral position, generated algorithmically using MATLAB. The geometry of the leaflet was determined by a combination of six parameters, as shown in Table 1 and Figure 2.

**TABLE 1:** Summary of key parameters for PHV design.

No	Parameter	Description	Range
1	HGT	Base height of the leaflet	10—25 mm
2	DIA	Standardized diameter of the prosthesis, intended for the desired size of the leaflet	15—40 mm
3	THK	Uniform thickness of the leaflet	0.1—1.0 mm
4	CVT	Radius of curvature of the leaflet belly, where 0 represents a flat leaflet and 1 represents the maximum possible curvature given the selected parameters	0–1
5	ANG	Elevation or depression angle of the leaflet free edge	−30°—+30°
6	ELM	Young’s modulus of the material used for the leaflet	0.5—20 MPa

By varying the values of these parameters, within specified ranges, the MATLAB algorithm generated a set of lines, a surface, and finally an STL mesh composed of geometric primitives (triangles). All models are represented as shells, described by 1,109–10464 S3-type elements per leaflet, aligning well with the approach endorsed in modern scientific approaches (Johnson et al., 2021). A total of 11,565 leaflet designs were generated.

### Data collection

The models were analyzed using FEM to simulate the effect of pressure on the leaflet opening. Within the MATLAB environment a calculation file for “Abaqus/CAE” was created, specifying the following:

• Boundary conditions. The valve model was fixed to prevent displacement along the lower edge (“encastre” boundary condition) in all six degrees of freedom. This was done to mimic the method of attachment of the valve to the prosthetic valve.
• Areas of pressure influence. A physiological pressure corresponding to the pressure in the aortic root was applied to the leaflet outlet surface, while a pressure corresponding to the pressure in the ventricle was applied to the leaflet inlet surface. The dynamic pressure changes were consistent with literature data (Pfensig et al., 2017). However, considering that only the opening phase was simulated, we selected a limited duration of pressure application, 0.2 s, during which the leaflets open.
• Modeling settings. We used a simplified material model that linearly described the biomechanics of the valve leaflet based on the elastic modulus. Although such a material model simplifies the behavior of the blade, this approach should be consistent for comparative analysis and determination of optimal geometries. In total, we utilized 20 material models that have the potential for use in the design of a valve apparatus. A detailed list of materials and their elastic moduli is provided in Supplementary Appendix SA.

The results of all 11,565 numerical experiments were then systematically analyzed using our Python algorithm. We note that this work represents a proof-of-concept approach to semi-automated optimization; therefore, at this stage, we deliberately limit our analysis to the most important parameters from our perspective: the opening area (LMN) and the maximum principal stress (STS). The former is a primary indicator of prosthetic performance and illustrates the effects of optimization. The latter serves as a key criterion for leaflet material failure under sustained loads (Claiborne et al., 2013; Chen et al., 2019), leading us to focus primarily on optimizing the mechanical stress distribution.

All valve design generation, FEM, and data collection processes were performed simultaneously on three high-performance computers, each equipped with a 4-core, 8-thread Intel Core i7-3770K processor with 3.4 GHz base clock frequency, 32 GB of RAM, and an Nvidia Quadro RTX 4000 graphics processor with 8 GB of video memory. The average wall clock time for each calculation and analysis was 766 s (12.8 min).

### Application of machine learning in generative design

We use six parameters–HGT, DIA, THK, CVT, ANG, and ELM - as features to train our model with two parameters, LMN and STS, used as targets. Our choice of ML algorithms was motivated through an extensive research into applied techniques within the medical domain, including Decision Tree (Quinlan, 1986), Random Forest (Breiman, 2001), Extra Tree (Geurts et al., 2006), Neural Network (Hornik et al., 1989), eXtreme Gradient Boosting (XGBoost) (Chen and Guestrin, 2016), Light Gradient Boosting Machine (Light GBM) (Ke et al., 2017), CatBoost (Prokhorenkova et al., 2017), and Ensemble (Valentini and Masulli, 2002) with the addition of Stacking (Wolpert, 1992). These regressors are integrated into a single AutoML framework for training and validation. In addition, we used a “Baseline” regressor, which makes predictions by averaging the target from training data, and acts as a comparison point for the 8 regressors evaluated but is not used for PHV prototyping.

Normalization can significantly influence the performance of ML algorithms, prompting us to investigate how different normalization techniques influence input parameters and model output. We applied normalization separately on input (geometric parameters of a PHV design) and model output, focusing on 5 types of normalization: 1) *Raw*–no transformation is applied, 2) *MinMax*–features are transformed by scaling each feature to a given range (from 0 to 1), 3) *Standard*–features are standardized by removing the mean and scaling to unit variance, 4) *Robust*–features are scaled using statistics that are robust to outliers, removing the median and scaling according to the quantile range, 5) *Power*–a family of parametric, monotonic transformations that make data more Gaussian, using the Yeo-Johnson transformation (Yeo and Johnson, 2000) to optimize variance and minimize skewness.

Our study consist of 11,565 designs with 9,252 designs randomly selected for training and 2,313 designs for validation. Since the focus of this study is the prediction of numerical scores (regression), Root Mean Square Error (RMSE) was used as the primary loss function. We implemented and evaluated two single-output ML models, with the first model predicting LMN scores and the second model predicting STS scores.

We applied several high-quality techniques to optimize the performance of our ML model during training. These techniques include *golden features*, *k-means features*, *feature selection*, *hill climbing*, and *boost on errors*, which have demonstrated their effectiveness in various areas of ML.

*Golden features* are a set of features with high predictive power. They are generated by applying mathematical operators (e.g., summation) to pairs of original features. To identify the best features, we generated all possible unique pairs of original features and randomly subsampled them to 250,000 if there were more than 250,000 pairs. The predictive power of a newly created feature was estimated using the decision tree algorithm with a maximum depth of 3. Only the top 10 new features were added to the training data as golden features.

*K-means features* are created using K-means clustering, which adds features based on cluster separability. These features can include a sample’s distance from all cluster centroids and a sample’s cluster label.

The *feature selection* is done in two steps. First, a random feature with a uniform distribution between 0 and 1 is inserted into the data set. Subsequently, all irrelevant features are discarded. If an existing feature has an importance lower than the random feature added in the first step, it is dropped.

*Hill climbing* is a local search algorithm that continuously moves in the direction of increasing height to find the top of the mountain or the best solution to the problem (Hernando et al., 2018). It stops when it reaches a peak value where no neighbor has a higher value. Hill climbing is a variant of the generate and test method that generates feedback to decide which direction to move in the search space. Since the hill climbing algorithm is a greedy approach, its search moves in the direction that optimizes the cost (loss function). Note, however, that this algorithm does not backtrack the search space because it does not remember previous states.

Finally, *boost on errors* is an approach similar to boosting techniques where a future model is improved using past errors.

The models were trained on a computer system equipped with an Intel Core i9-10940X processor clocked at 3.30 GHz, an NVIDIA GeForce RTX 3090 graphics card, 256 GB of RAM, and the Windows 10 operating system.

### Exploration of optimal designs

To determine the optimal geometries of PHV, we implemented six state-of-the-art optimization algorithms for sampling geometric parameters and efficiently pruning suboptimal designs: Random Search (RS) (Bergstra and Bengio, 2012), Tree-structured Parzen Estimator (TPE) (Bergstra et al., 2011; Bergstra et al., 2013), CMA-ES-based Algorithm (CMA) (Auger and Hansen, 2005; Ros and Hansen, 2008; Hansen, 2016; Nomura et al., 2021), Nondominated Sorting Genetic Algorithm (NSGA) (Deb et al., 2002), Multiobjective Tree-structured Parzen Estimator (MOTPE) (Ozaki et al., 2022), and Quasi-Monte Carlo Algorithm (QMC) (Bergstra and Bengio, 2012). To efficiently perform the design search procedure, we implemented a pruning mechanism based on the Hyperband pruner (Li et al., 2017), which demonstrated its performance in various optimization experiments (Ozaki et al., 2020) and outperformed benchmarks with Kurobako (Imamura, 2020) for non-deep learning tasks. In our study, each optimization algorithm was limited to 2000 iterations.

To evaluate the geometric design parameters, we introduced our own objective function based on the weighted harmonic mean, allowing for direct estimation of lumen and stress scores for each PHV design. Each PHV geometry is evaluated by the design score DSN :

$$DSN=\frac{\left(1+\alpha^{2}\right)*{LMN}_{s}*{STS}_{s}}{\alpha^{2}*{{STS}_{s}+LMN}_{s}}$$ (1)

$${LMN}_{s}=LMN=\frac{ORFC}{{ORFC}_{\max}}$$ (2)

$${STS}_{s}=\left\{\begin{array}{c} 1-\frac{STS}{UTS},when\;\left(\frac{STS}{UTS}\right)<1\\ 0,when\;\left(\frac{STS}{UTS}\right)\geq 1 \end{array}\right.$$ (3)

where LMN and STS represent the absolute lumen and stress values obtained for a given set of geometric parameters in a design. α is a positive factor that allows for a trade-off between ${LMN}_{s}$ and ${STS}_{s}$ , with a value chosen such that ${LMN}_{s}$ is considered α times as important as ${STS}_{s}$ . Five different values of α were estimated, including 0.2, 0.5, 1.0, 2.0, and 5.0 ^1^. Ultimate tensile strength, UTS , represents the maximum stress a material can withstand before breaking when stretched or pulled. During the optimization phase, twenty UTS values were used, ranging from 2.34 to 57.1, corresponding to common materials used in medical device prototyping. ORFC and ${ORFC}_{\max}$ are the effective and geometric orifice areas, respectively. ORFC is calculated based on FEM modeling, while ${ORFC}_{\max}$ is equal to $\pi r^{2}$ , where r is the radius of the PHV. We note that LMN is equal to ${LMN}_{s}$ , which is obtained directly from trained models. This is due to the fact that LMN does not require further scaling as it falls within the interval used for quantitative design comparison. To ensure that design comparisons are unambiguous, all design scores, DSN , ${LMN}_{s}$ , and ${STS}_{s}$ , were normalized in the range of $\left[0,1\right]$ .

To verify the effectiveness of the algorithms in determining the optimal geometry of a designed device, numerical modeling was utilized for performance evaluation. This process involved selecting 500 random combinations of geometric parameters (100 geometries per each α value) from the datasets obtained during optimization and using ML models to predict LMN and STS values. These combinations were then analyzed by numerical modeling using the method outlined in “Data Collection”. The criterion for accuracy, in this case the predicted versus modeled results, was determined by calculating RMSE.

When examining differences between samples, such as predicted vs. modeled, a paired *t*-test was employed as the samples were considered dependent and used a common set of geometric parameters. Differences were considered reliable at a significance level of *p* < 0.05. The analysis was performed using the Python library “SciPy”, which specializes in statistical processing.

## Results

### Initial data analysis

**FIGURE 3:**
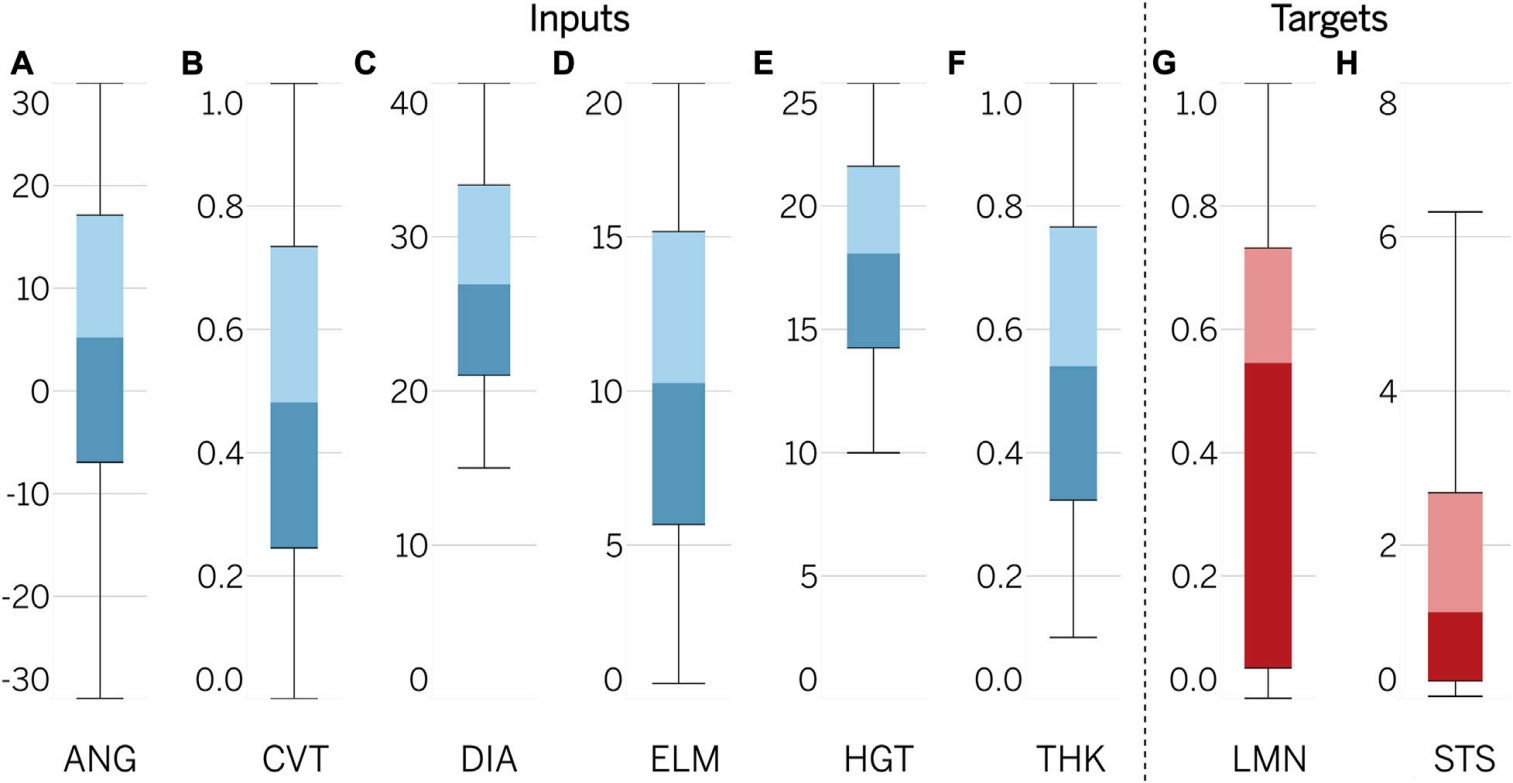
Distribution of input variables and output targets.

The dataset utilized for training consists of combinations of geometric parameters within predefined ranges, in addition to their corresponding results obtained through the Abaqus/CAE simulation environment. The distribution of the features, including HGT, DIA, ANG, CVT, THK, and ELM, was determined to be uniform due to the random nature of their generation, as depicted in Figure 3. Comparatively, the targets in the dataset were represented by shifted ranges. The median for LMN was 0.55, with an interquartile range (IQR) of 0.05–0.73 and a minimum-maximum range of 0.002–0.999. For STS, the median was 1.13, with an IQR of 0.23–2.67 and a minimum-maximum range of 0.04–12.81. Analysis of the data set revealed the existence of highly effective designs, with 175 of them having LMN values greater than 0.95. It is worth noting that 25% of the total dataset (2,892) failed to exceed 0.05 in terms of LMN. A similar pattern was observed for STS, with the majority of the sample represented by small values; specifically, 5,496 results did not exceed 1.0 MPa. However, there were also instances of high STS values, with 512 geometries exceeding the threshold of 5.0 MPa.

### Evaluation of machine learning models

**FIGURE 4:**
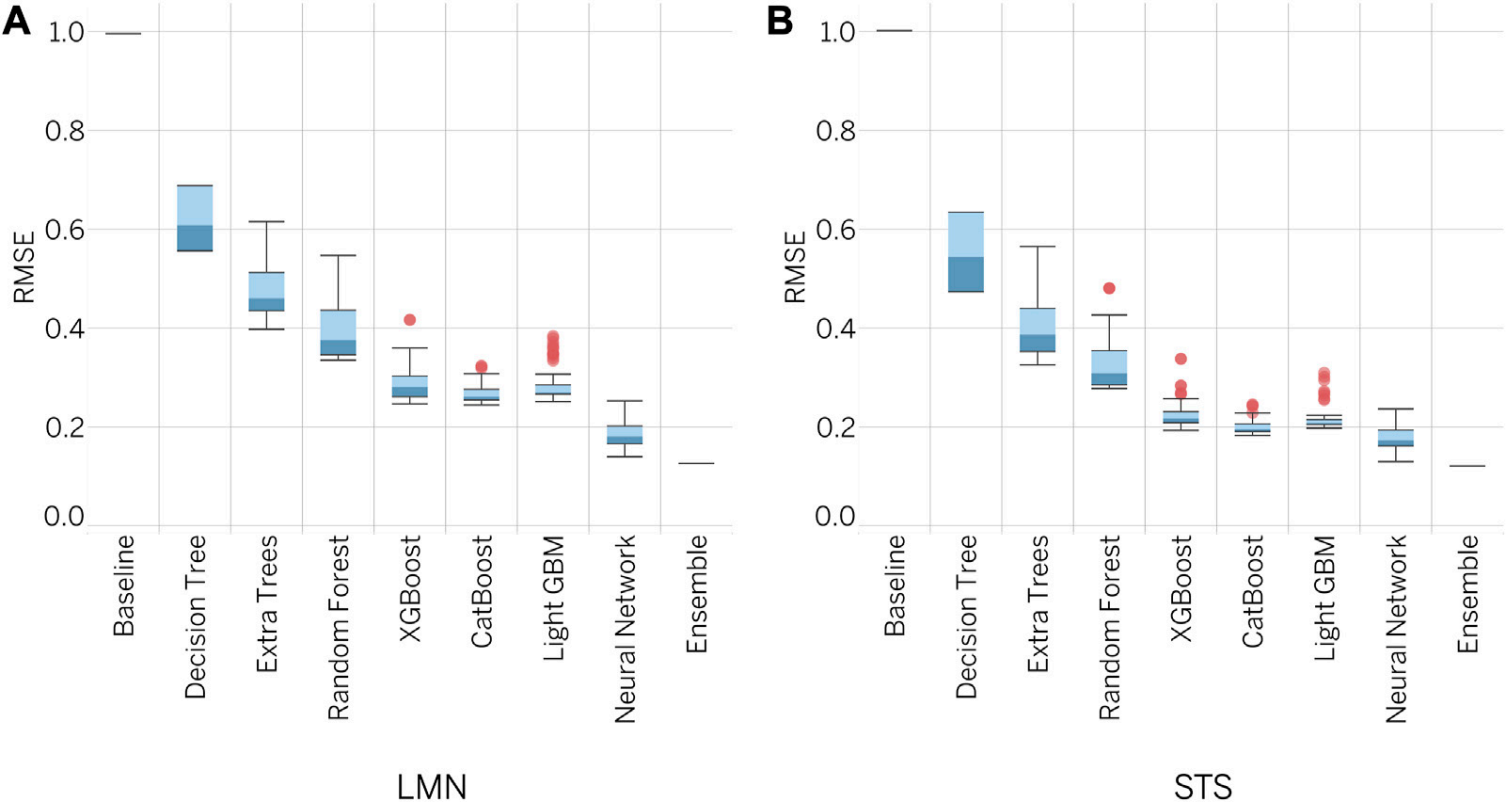
Performance of ML algorithms under study.

The AutoML framework was successfully implemented and used to train 341 models for LMN prediction and 347 models for STS prediction. The results indicate that the lowest RMSE was achieved by ensembles, with an RMSE of 0.126 for LMN and 0.121 for STS. Comparison with other models showed significant inferiority in terms of error, as illustrated in Figure 4. Further analysis and optimization efforts will focus on the use of ensemble-based models.

**FIGURE 5:**
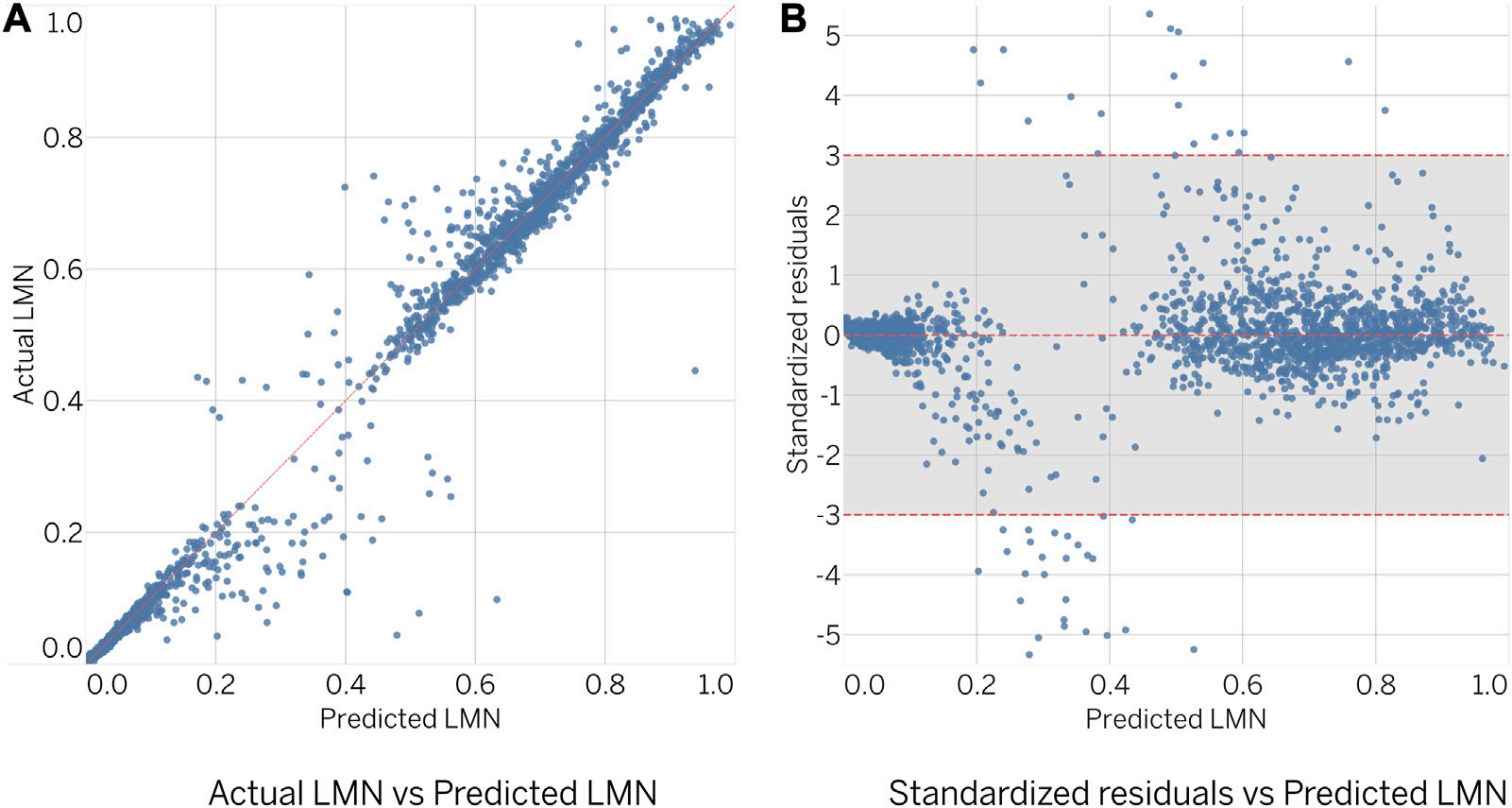
Comparison of actual and predicted values of LMN.

The predictive performance of the obtained ensembles on the validation subset was evaluated by comparing the predicted to actual values, as shown in Figure 5A, and by examining the standardized residuals, as shown in Figure 5B. Ideally, the results should be closely aligned with a regressed diagonal line (Figure 5A) or horizontal zero axis (Figure 5B). However, further analysis revealed a relatively large error for 64 out of 2,313 designs (2.8% of the total), exceeding the limits of three standard deviations. Despite this, statistical analysis showed no significant difference between actual and predicted samples ( p=0.151 ).

**FIGURE 6:**
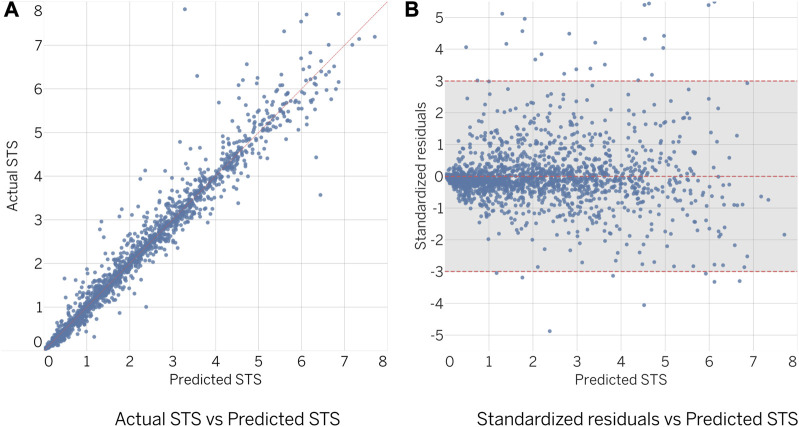
Comparison of actual and predicted values of STS.

The evaluation of the STS score (Figure 6) showed similar results to the LMN ensemble. However, the number of geometries exceeding the three standard deviation threshold was lower, with 35 out of 2,313 designs (1.5% of the total) observed. Statistical analysis revealed no significant difference between the actual and predicted samples ( p=0.092 ).

To formally evaluate the performance of the ensembles, various metrics were estimated on the training and validation subsets. Table 2 presents a summary of the results, including five error-based metrics that provide an average measure of the proximity of the predictions to the expected values. Furthermore, Supplementary Appendix SB provides a comprehensive set of regression metrics that fully describe the ensembles obtained and the distribution of RMSE across different ML algorithms used during training. Detailed descriptions and equations for these metrics can be found in the publicly available project repository ^2^.

**TABLE 2:** Evaluation of model performance on training and validation subsets.

No	Metric	LMN		STS	
		Train	Validation	Train	Validation
1	MAPE	0.116	0.118	0.093	0.102
2	WAPE	0.042	0.044	0.077	0.081
3	MAE	0.018	0.019	0.127	0.135
4	NRMSE	0.040	0.044	0.022	0.029
5	R^2^	98.7%	98.4%	97.0%	96.8%

In recent years, the use of ML models has become increasingly widespread, but the interpretability of these models remains a significant challenge. One method that has been proposed to increase the transparency and interpretability of ML models is the SHapley Additive exPlanations (SHAP) method (Lundberg et al., 2017). SHAP scores are based on cooperative game theory and have been widely used to understand the main features that affect the performance of a given model.

**FIGURE 7:**
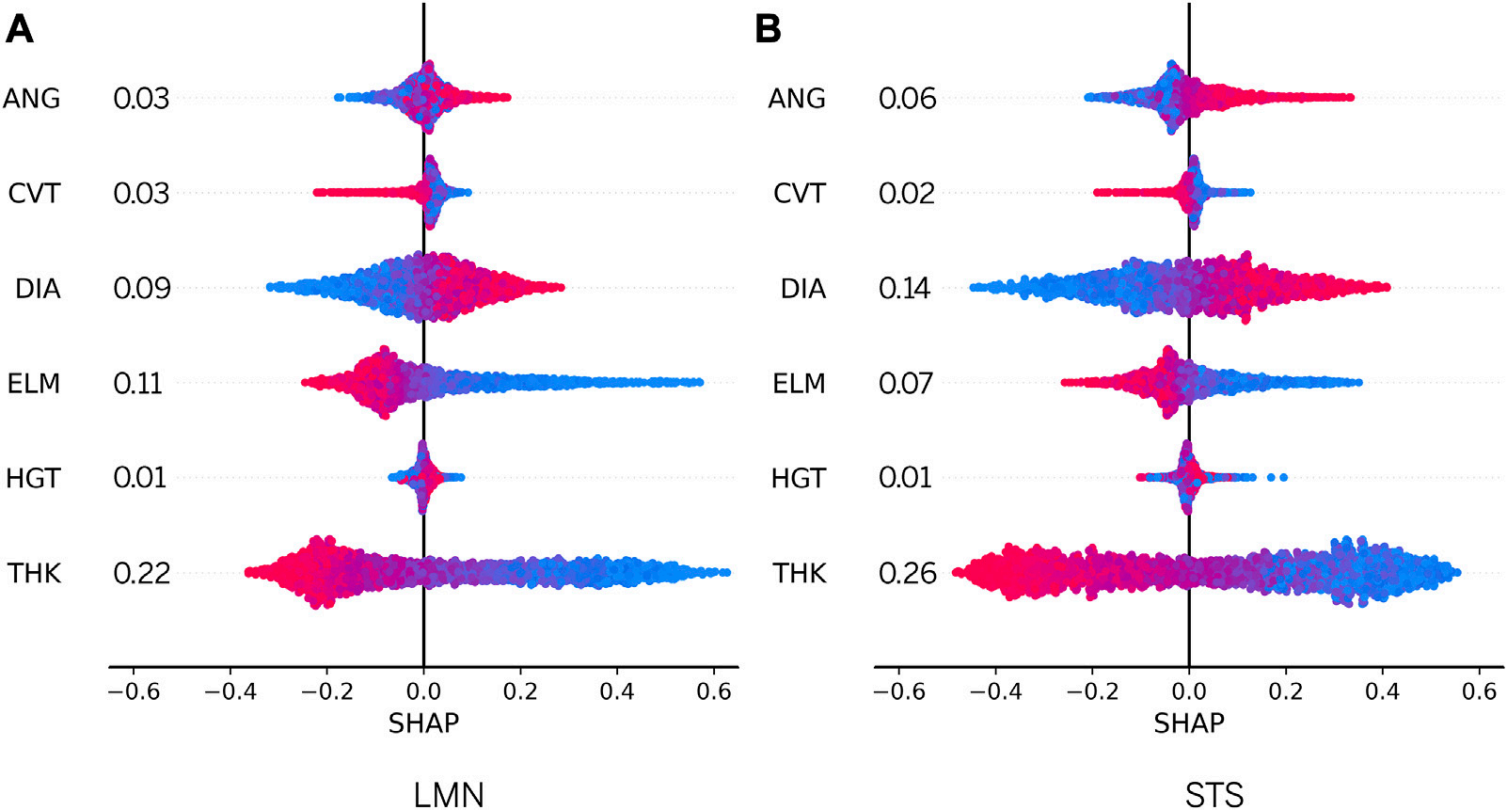
Estimation of SHAP values for model features. All instances are drawn using the blue-violet-red color scale. Red and blue dots represent features with high and low values respectively, while violet represents intermediate feature values.

In this study, we applied SHAP to evaluate feature importance of two ensembles using a validation subset. To provide a clear visualization of the results, we employed a bee swarm plot, which is designed to display an information-dense summary of how top features in a dataset affect the model’s output. In the bee swarm plot, each PHV geometry in the given explanation is represented by a single dot on each feature row. The X position of the dot is determined by the SHAP value of that feature, and dots “pile up” along each feature row to show density. In addition, a blue-purple-red color scale was used to display the original feature values. It is important to note that while the SHAP values provide insight into the contribution or importance of each feature to the model’s prediction, they do not evaluate the quality of the prediction itself. The results of our analysis are shown in Figure 7.

After conducting an analysis of the swarm plots for the LMN and STS ensemble models, our findings indicate that both models have relatively similar feature importance. Specifically, DIA and ANG were found to have a positive contribution to the prediction output when their values were high, and a negative contribution when their values were low. Conversely, THK and ELM were found to have a positive contribution to the prediction output when their values were low, and a small negative contribution when their values were high. Furthermore, CVT was found to have a weak prediction contribution whilst HGT had almost no contribution in both ensemble models. The global feature importances are depicted in the feature rows in Figure 7. For further analysis, the distribution of SHAP values for each feature is presented in Supplementary Appendix SC.

### Evaluation of generated PHV designs

We used a Hyperband-based pruning mechanism in conjunction with six optimization algorithms to identify optimal PHV geometries. Here, the search space of explored parameters was strictly defined to match modern manufacturing standards, to ensure applicability in medical device manufacturing, as described in Table 1, and omit parameter values that are unfeasible in a real setting.

**FIGURE 8:**
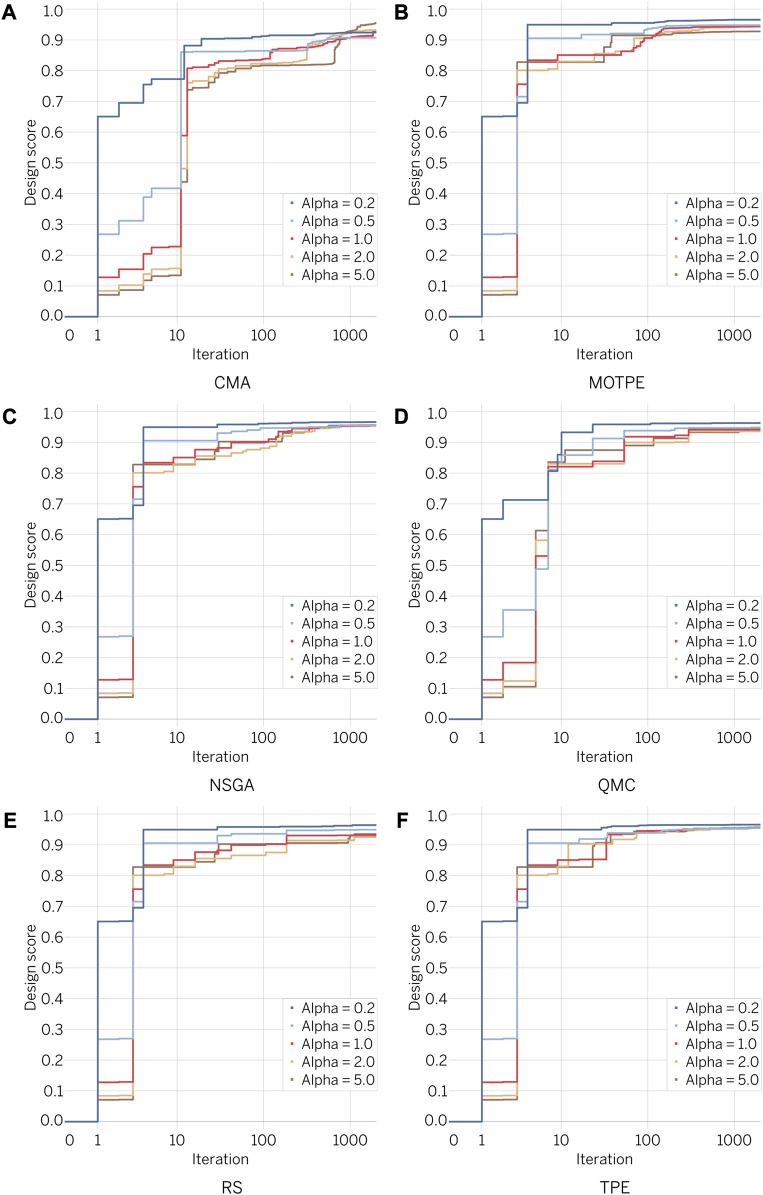
Design score dynamics for different optimization algorithms.

Figure 8 showcases the results from optimal design exploration. Each optimizer performed 2000 iterations per alpha value, using five different alpha values: 0.2 and 0.5 (optimization prioritizing STS); 1.0 (balanced optimization between LMN and STS); 2.0 and 5.0 (optimization prioritizing LMN). In total, each optimization algorithm evaluated 10,000 designs with different characteristics (2000 iterations × 5 alpha values). As shown in Figure 8, designs prioritizing STS (alpha values of 0.2 and 0.5) converged faster to higher design scores. In contrast, designs prioritizing LMN converged more slowly. Balanced designs (alpha value of 1.0) showed intermediate convergence dynamics whilst the best designs studied converged to a similar range of scores, with DSN above 0.90.

As part of our optimal design exploration, we evaluated two methods for assessing the importance of hyperparameters: Mean Decrease Impurity (MDI) and Functional ANOVA (fANOVA) (Hutter et al., 2014). Both methods use a random forest regression model to predict the objective value based on a given parameter configuration. The accuracy of this model directly affects the reliability of the importance scores provided by the evaluators. For both evaluators, the number of trees in the forest was set to 64, and the maximum depth of the trees was set to 64.

**FIGURE 9:**
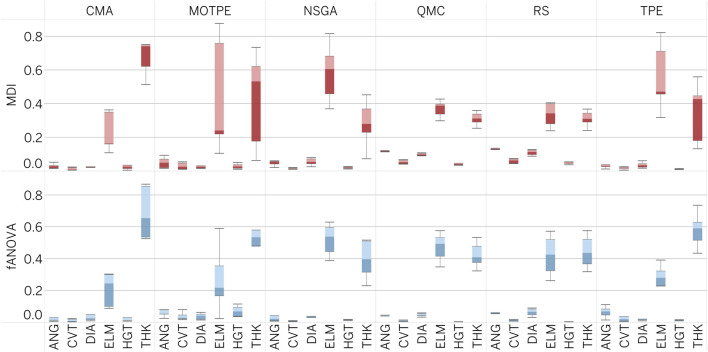
Importance of geometry parameters as computed by MDI and fANOVA.

The results of the feature importances, as depicted in Figure 9, indicate that MDI and fANOVA results are similar in nature to those of SHAP. All optimization algorithms consider Young’s modulus (ELM) and PHV thickness (THK) as the most influential parameters with a significant impact on the optimization process. In contrast, four other geometry parameters including ANG, CVT, DIA, and GHT were found to have little effect.

### Finite element analysis of generated PHV designs

The quantitative evaluation on the test subset is of utmost importance during the *in-silico* verification of the optimal designs. The results, as presented in Table 3, indicate that the metrics for the geometries obtained during the test phase are comparable to the values obtained during the training and validation phases. A comprehensive set of metrics designed to assess the accuracy of LMS and STS predictions for the three sample groups (training, validation, and test) is detailed in Supplementary Appendix SB.

**TABLE 3:** Evaluation of model performance on *in silico* test subset.

No	Metric	LMN	STS
1	MAPE	0.163	0.086
2	WAPE	0.032	0.074
3	MAE	0.018	0.075
4	NRMSE	0.041	0.021
5	R^2^	98.6%	95.5%

The optimization algorithms themselves have demonstrated remarkable performance by selecting geometric parameters of the leaflet that result in high DSN scores. Despite a shift in the balance of optimality towards either LMN or STS, all algorithms were able to identify geometries with high integral DSN scores in the range of 0.86–0.97.

**FIGURE 10:**
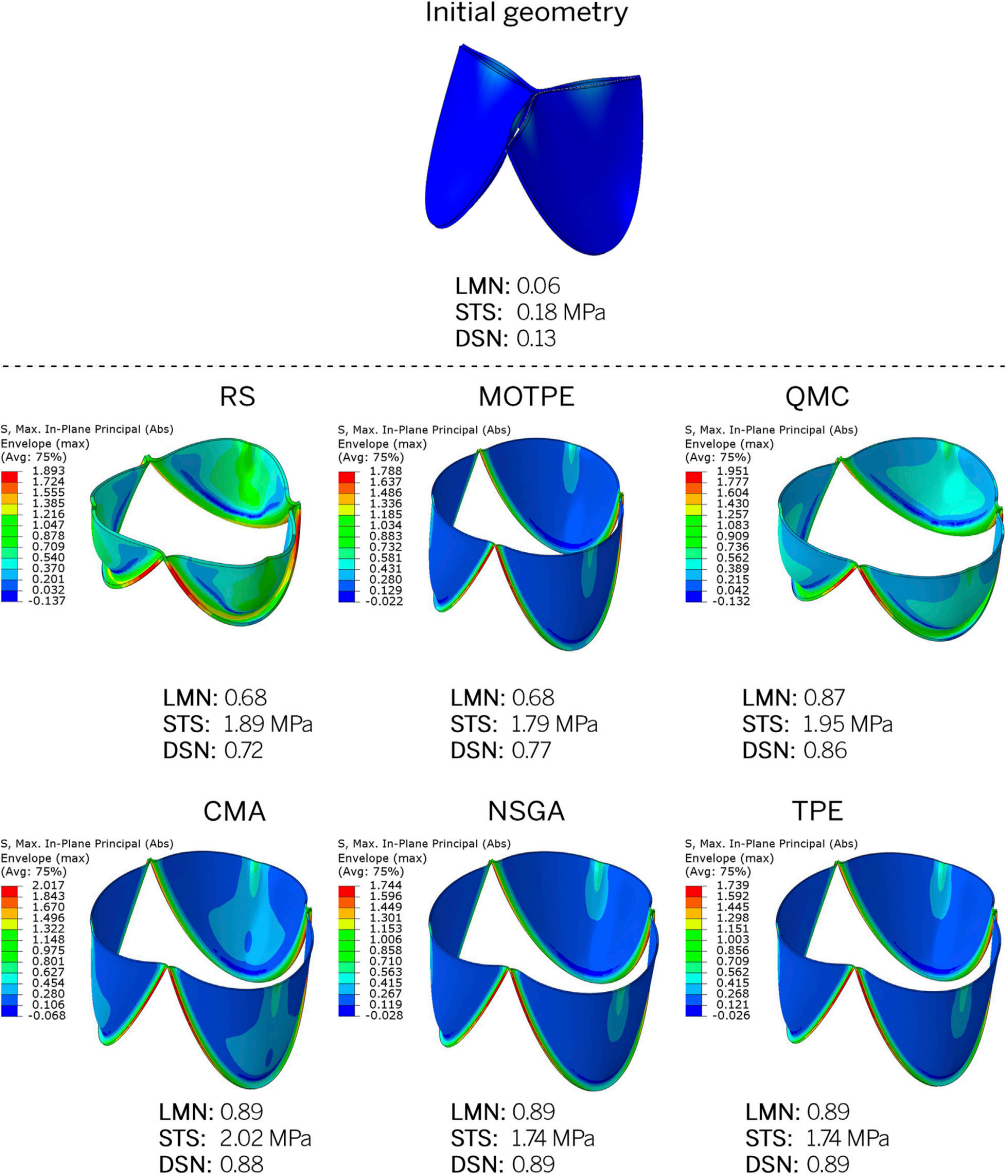
Examples of final designs resulting from the studied optimizers: epiphyses in the open state after pressure application simulation. The initial geometry from which all algorithms started optimization is also shown.

A qualitative examination of the optimization results confirms the efficiency of the algorithms. All algorithms effectively selected parameter combinations that resulted in optimal leaflet opening while minimizing stress, as demonstrated in Figure 10 and Supplementary Appendix SD. The optimal leaflet models typically exhibit high LMN opening areas while maintaining moderate STS values, which in most cases do not exceed 2.0 MPa. This is well below the strength limit of the material models. It is noteworthy that the optimization algorithms show similar trends in selecting the best geometric indicators. The CMA, NSGA, QMC, and TPE algorithms selected larger diameters (31.0–32.2 mm) as optimal, minimized the leaflet lift angle for a better opening, and showed a similar thickness (0.30–0.34 mm). However, the parameters of HGT and CVT varied significantly among the optimizers and no general trend was observed.

## Discussion

The design of PHV leaflets plays a critical role in ensuring both their efficacy and longevity (Sebastian et al., 2019; Xuan et al., 2020). Nevertheless, these materials are susceptible to degradation and calcification, which can lead to valve malfunction (Kostyunin et al., 2020). A major contributor to biomaterial degradation is cyclic mechanical stress (Claiborne et al., 2013; Chen et al., 2019). Therefore, it is imperative to optimize leaflet design based on the distribution of mechanical stress. This is precisely why numerical simulation has emerged as an essential foundation for such optimization, becoming a crucial element in the advancement of medical device research (Bologna et al., 2023) and the assessment of stress-strain patterns in specific pathological conditions within the cardiovascular system (Di Giuseppe et al., 2021). FEM is particularly advanced among numerical simulation tools and has been demonstrated to be highly efficient in modeling PHVs (Borazjani, 2013; Gilmanov and Sotiropoulos, 2016; Castravete et al., 2020; Lee et al., 2020; Chen et al., 2022). Furthermore, existing research has demonstrated advanced optimization algorithms that enable the semi-automatic generation and FEM analysis of significant quantities of PHVs, facilitating the selection of optimal candidates among them (Hsu et al., 2015; Li and Sun, 2017; Abbasi and Azadani, 2020; Gulbulak et al., 2021). Among the most sophisticated approaches in this context, we consider the work of Travaglino et al. (Travaglino et al., 2020). This study developed a computational framework using a Bayesian algorithm to optimize leaflet geometry in transcatheter aortic valves. The authors used a specialized machine learning tool to guide the optimization process, rather than exhaustively enumerating potential leaflet configurations to identify the optimal solution. They employed a combination of spline parameters and FEM based on bovine and porcine pericardial material models to investigate approximately 1,000 leaflet designs under nominal circular deployment and physiological loading conditions. The optimal parameter values for the valve model were obtained, resulting in leaflet shapes that reduced peak stress by approximately 17% compared to the initial model.

Our study builds upon this concept showcasing the application of six state-of-the-art optimization algorithms. These algorithms not only automate the selection of optimization directions, but also lead to the generation of the most appropriate geometries. While these algorithms are widely represented in ML hyperparameter search problems, their application to the optimization of leaflet devices is novel and constitutes a primary focus of our investigation. These algorithms differ in how they explore and exploit the search space, balance the trade-off between exploration and exploitation, and handle multiple objectives and constraints. RS is a simple algorithm that randomly samples parameters from a uniform distribution without using any information from previous evaluations. TPE is a Bayesian optimization algorithm that models the probability of improvement as a function of parameters and uses a tree structure to adaptively partition the search space. CMA is an evolutionary algorithm that adapts the covariance matrix of a multivariate normal distribution to generate new solutions. NSGA is a genetic algorithm that uses a non-dominated sorting procedure to rank solutions according to their Pareto dominance, and a crowding distance measure to maintain diversity. MOTPE is an extension of TPE for multiobjective optimization that uses a hypervolume indicator to guide the search toward the Pareto front. QMC is an algorithm that uses low-discrepancy sequences to sample parameters more uniformly than random sampling, and can achieve faster convergence rates than RS. These algorithms have been applied to various problems in geometry and device optimization, such as shape, design and process optimization.

Our results showed that NSGA and TPE were the most effective optimizers for this task. Of note, TPE successfully achieved the desired result in less than 100 iterations, establishing these algorithms as the preferred choice for addressing similar challenges. NSGA is a widely used genetic algorithm that guarantees the inclusion of individuals with extreme values of the target functionals in the set of parents (Joshi et al., 2021; Rego et al., 2022; Saravanamuthu et al., 2022). TPE, on the other hand, is a Tree-Structured Parzen Estimator algorithm typically used to optimize ML model hyperparameters (Bergstra et al., 2011; 2013), and it was intriguing to find that it was also suitable for optimizing a heart valve design. In contrast, the Random Search algorithm performed the worst, as expected due to its limited number of iterations.

Furthermore, in the presented sequence of “generation - modeling - optimizer”, we incorporated an element of surrogate FEM based on ML, which aims to accelerate the execution of numerical calculations, which becomes a bottleneck in cases with thousands of geometries in FEM. Such an approach for predicting the stress-strain state of the leaflet and its coaptation area (Balu et al., 2019) or the geometric orifice area (Gulbulak et al., 2021) has already demonstrated its validity. In this study, FEM modeling of valve biomechanics has been adapted through the integration of ML techniques. The methodology involves the use of an algorithm to predict the peak stress experienced by the valve leaflet, coupled with ML evaluation of the geometric opening area of the valve when it is in the fully open state in a prosthetic heart valve. This is done with a primary optimization focus of increasing the leaflet opening area.

Our ML models were trained on a large sample of geometries and materials, including animal pericardium and synthetic polymers, to enable the optimization of the prosthetic heart valve with different leaflet materials. Unlike previous studies that focused on PHV performance measured by hydrodynamic efficiency and durability (Liang and Sun, 2019; Travaglino et al., 2020), we employed ML models with RMSE less than 13% and $R^{2}$ values greater than 0.96. A similar study by Balu et al. (Balu et al., 2019) introduced the concept of deep learning based on FEM analysis to study the biomechanics of aortic valve bioprosthesis deformation, achieving a valve coaptation prediction efficiency of $R^{2}=0.87$ . In contrast to our study, their distribution of the “predicted vs. true” plot did not show a clear division into two regions.

Our study included modeling the opening of the PHV, which resulted in the data being split into two states, corresponding to the open state and the case where the PHV failed to open (for example, if the material was too stiff). In another study, Liang and Sun presented a proof-of-concept using deep learning to design artificial aortic valves and evaluated the qualitative and quantitative distribution of the stress field (for a closed valve) generated by the neural network compared with FEM data (Liang and Sun, 2019). The results showed a maximum discrepancy of 4.1% between FE-calculated and ML-predicted stress. However, it is important to note that this comparison was not made over a wide range of parameters with extreme values, as in our study. Overall, the prediction performance of the ML algorithm employed is considered comparable to the above-mentioned work. Furthermore, it was found that among the algorithms studied, ensemble methods exhibited the highest performance for both targets, surpassing the performance of tree-based, neural-network-based, and gradient-boosting-based algorithms. This superior performance is likely due to the complex and non-obvious dependencies between parameters and target metrics present in the dataset.

## Limitations

This study presents a proof-of-concept framework for optimizing valve leaflet geometry that is designed to showcase the capabilities of specialized machine algorithms and optimizers. As a result, certain constraints were deliberately imposed on FEM.

First, we focused exclusively on the opening phase of the valve. Literature shows that the highest stresses within the leaflet material occur during the diastolic phase, so it is rational to evaluate this factor throughout the cardiac cycle. However, in this work, one of our objectives was to maximize the opening area of the leaflet apparatus, thus limiting the scope of the *in-silico* investigations. Undoubtedly, future research should include the modeling of the entire cardiac cycle and the analysis of quantitative characteristics throughout its entirety.

Second, we deliberately excluded the prosthesis frame components from the FEM setup by using a linear material description. The purpose of such assumptions is to avoid being tied to a specific prosthesis design with its unique geometry and mechanical component properties. Comprehensive modeling of the entire construct would require implementing descriptions of support frame properties, which vary significantly in material composition, including metals, polymers (Mylotte et al., 2013), and others. Researchers and engineers wishing to apply this framework in practice could certainly augment the assembly FEM with all relevant prosthetic components. However, current work is intended to demonstrate the viability of the concept of using ML optimizers. Similar considerations apply to the description of leaflet materials - several models, especially for polymers, can be linearly approximated within small deformation ranges. Of course, biomaterials should ideally be described more comprehensively (Borazjani, 2013; Hsu et al., 2015). In this case, we deliberately simplified their description to linear for the sake of a unified problem formulation and due to the limitations of ML algorithms in handling complex models.

Third, the current numerical modeling setup does not incorporate the fluid (blood) domain and the corresponding fluid-structure interaction modeling (Borazjani, 2013; Hsu et al., 2015; Gilmanov and Sotiropoulos, 2016). This assumption was primarily motivated by the need for reasonable computational times, as the development of the algorithm involved the execution of 11,565 numerical experiments. Significant complication by FSI modeling would exponentially increase the computational cost, requiring either a reduction in the space of geometries to be analyzed or an impractical increase in computational time. The cumulative time spent on these experiments amounted to 103 machine days, a figure that would undoubtedly be multiplied many times over in the case of FSI. Nevertheless, the evolutionary path of the present framework towards the incorporation of FSI is well justified and promising, since the effects (mainly shear stress) that occur in the leaflet material during blood contact in the context of FSI critically affect its internal state.

Finally, future studies could potentially benefit from the use of a more advanced and fine-tuned computer modeling method, such as fluid-structure interaction. Despite these limitations, the objectives of the study were successfully achieved.

Certainly, all of the aforementioned limitations will significantly improve and refine the accuracy of the presented framework and thus become focal points for future work.

## Conclusion

The utilization of ML algorithms has been an area of growing interest in recent years due to their ability to automate complex intelligent tasks. Our research has demonstrated the feasibility of applying ML to optimize the geometry of prosthetic heart valve leaflets, providing a new, efficient, and productive approach. However, it was found that the performance of the coupling of the mesh generator, explorer, and optimizer is dependent on the chosen regressor model and optimizer model. The results of this study suggest that the use of an ensemble trained on FEM data in conjunction with a Genetic Algorithm-based (NSGA) or Tree-Structured Parzen Estimator-based (TPE) optimizer can effectively search for the optimal configuration of prosthetic heart valve leaflets. These findings have potential implications for the development of new medical device design methods using ML algorithms.

## Data Availability

The data collected in this study can be accessed via the GitHub repository at: https://github.com/ViacheslavDanilov/generative_design.

## Footnotes

1

α=0.2 or α=0.5 represent cases when ${STS}_{s}$ is considered 5 and 2 times as important as ${LMN}_{s}$ ; α=1.0 implies a perfect balance between ${LMN}_{s}$ and ${STS}_{s}$ ; α=2.0 or α=5.0 represent cases when ${LMN}_{s}$ is considered 2 and 5 times as important as ${STS}_{s}$ .

2
https://github.com/ViacheslavDanilov/generative_design/blob/main/tools/metrics.py.
