# Supplementary figures and images for: Perfect prosthetic heart valve: generative design with machine learning, modeling, and optimization
Source: Front Bioeng Biotechnol. 2023 Sep 15;11:1238130. doi: 10.3389/fbioe.2023.1238130 (PMC10541217; doi:10.3389/fbioe.2023.1238130)

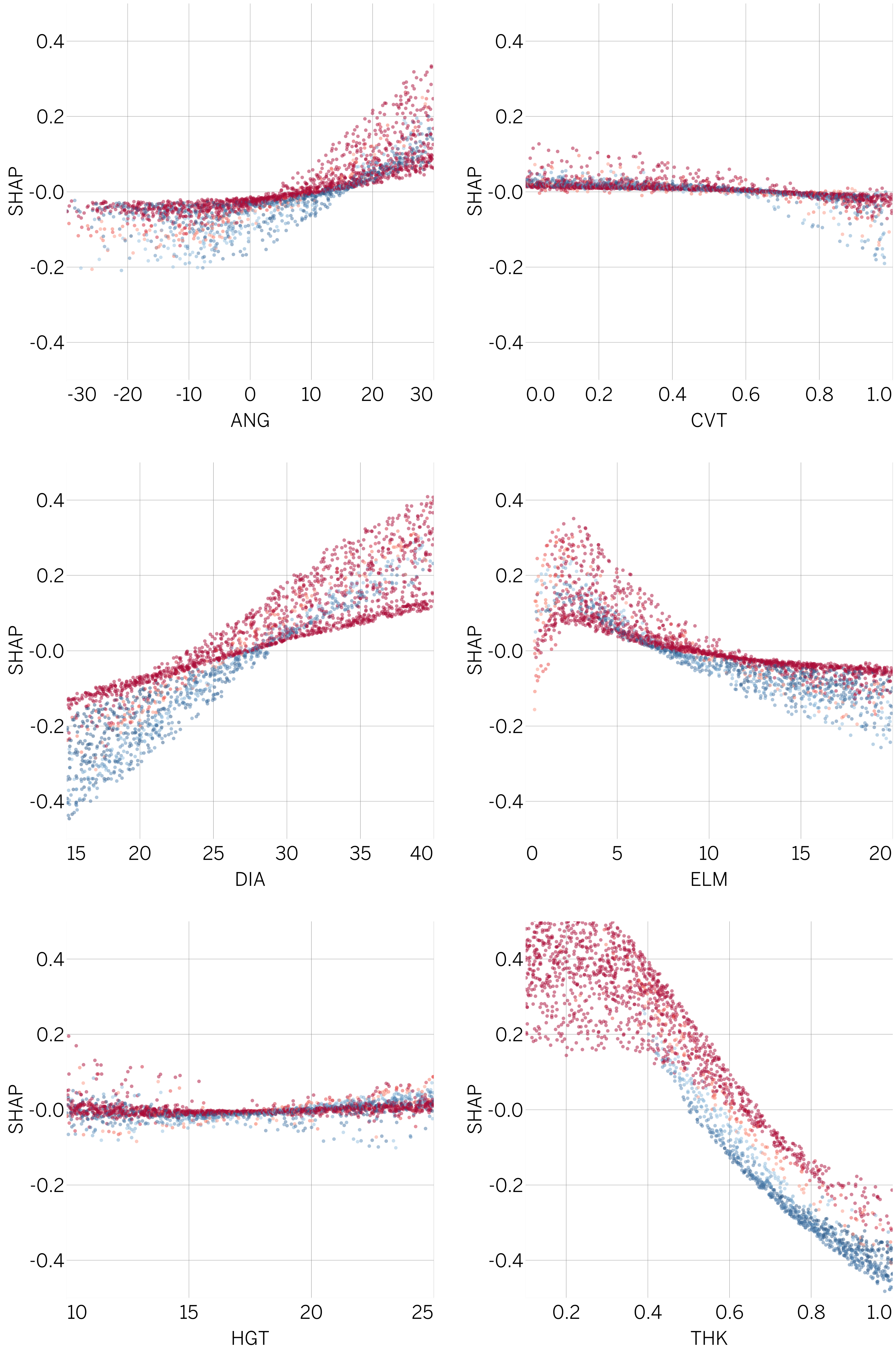

Supplement: Supplementary file 2 [file Image3.tif]

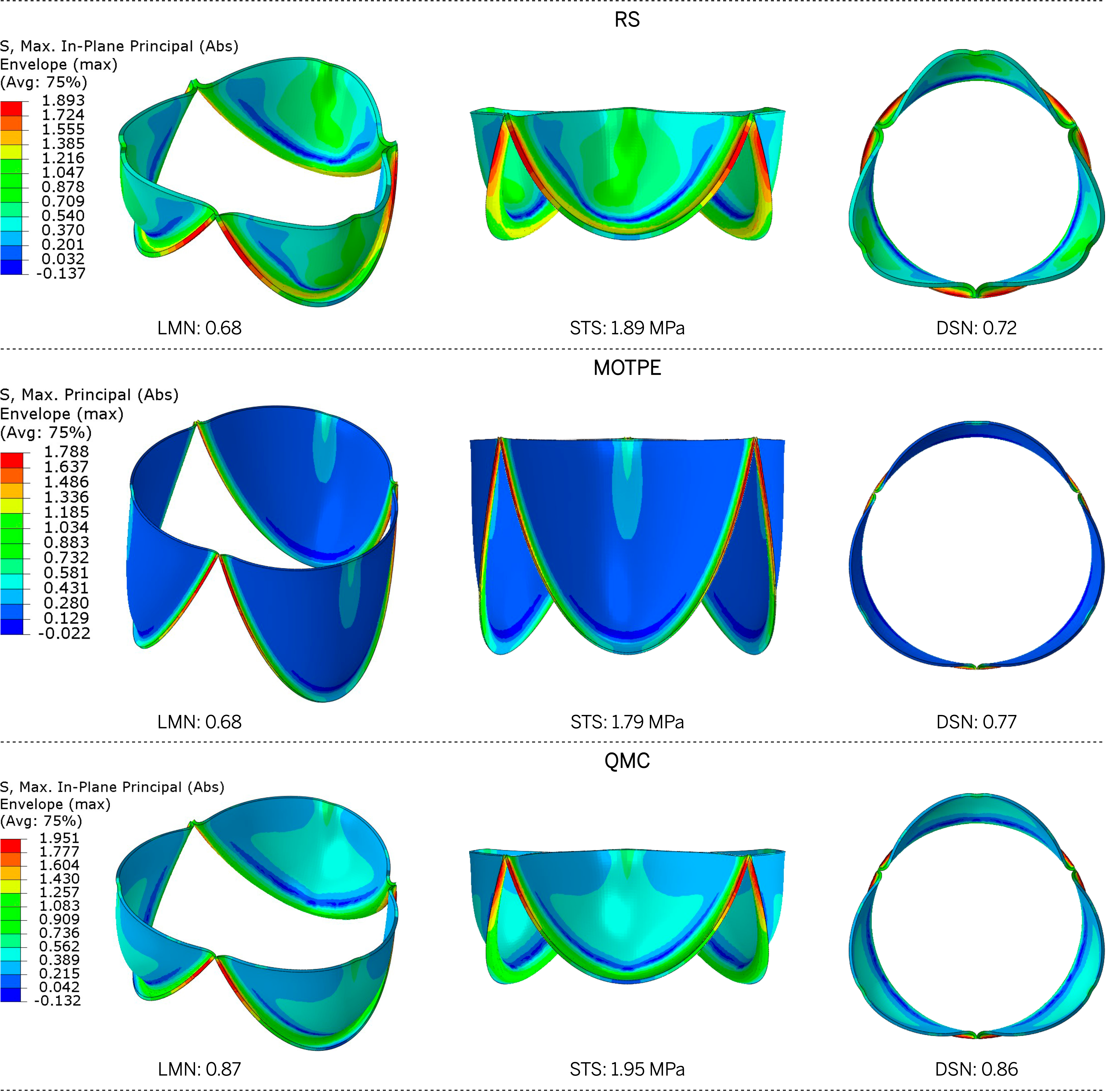

Supplement: Supplementary file 3 [file Image4.tif]

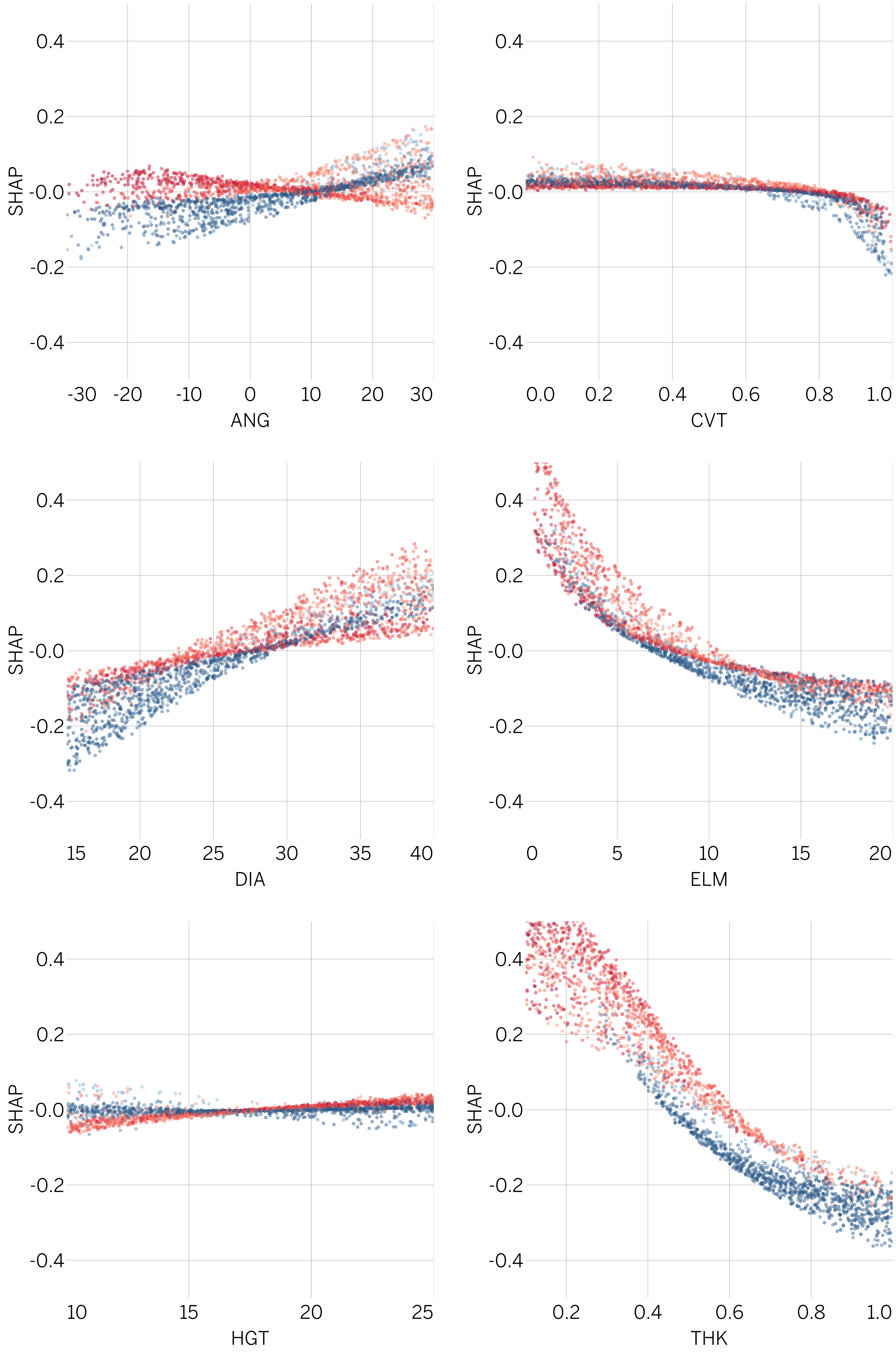

Supplement: Supplementary file 4 [file Image2.tif]

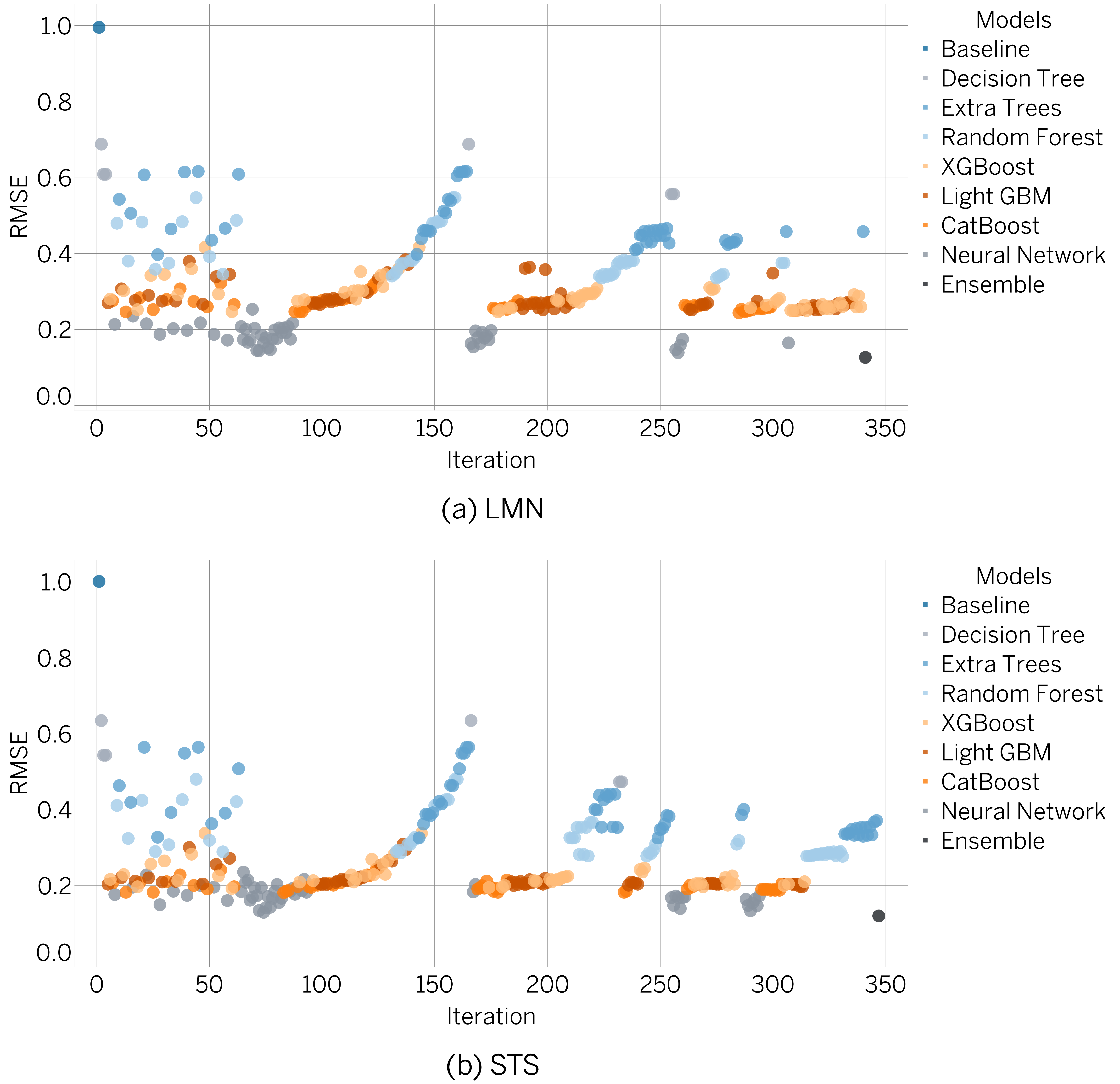

Supplement: Supplementary file 5 [file Image1.tif]

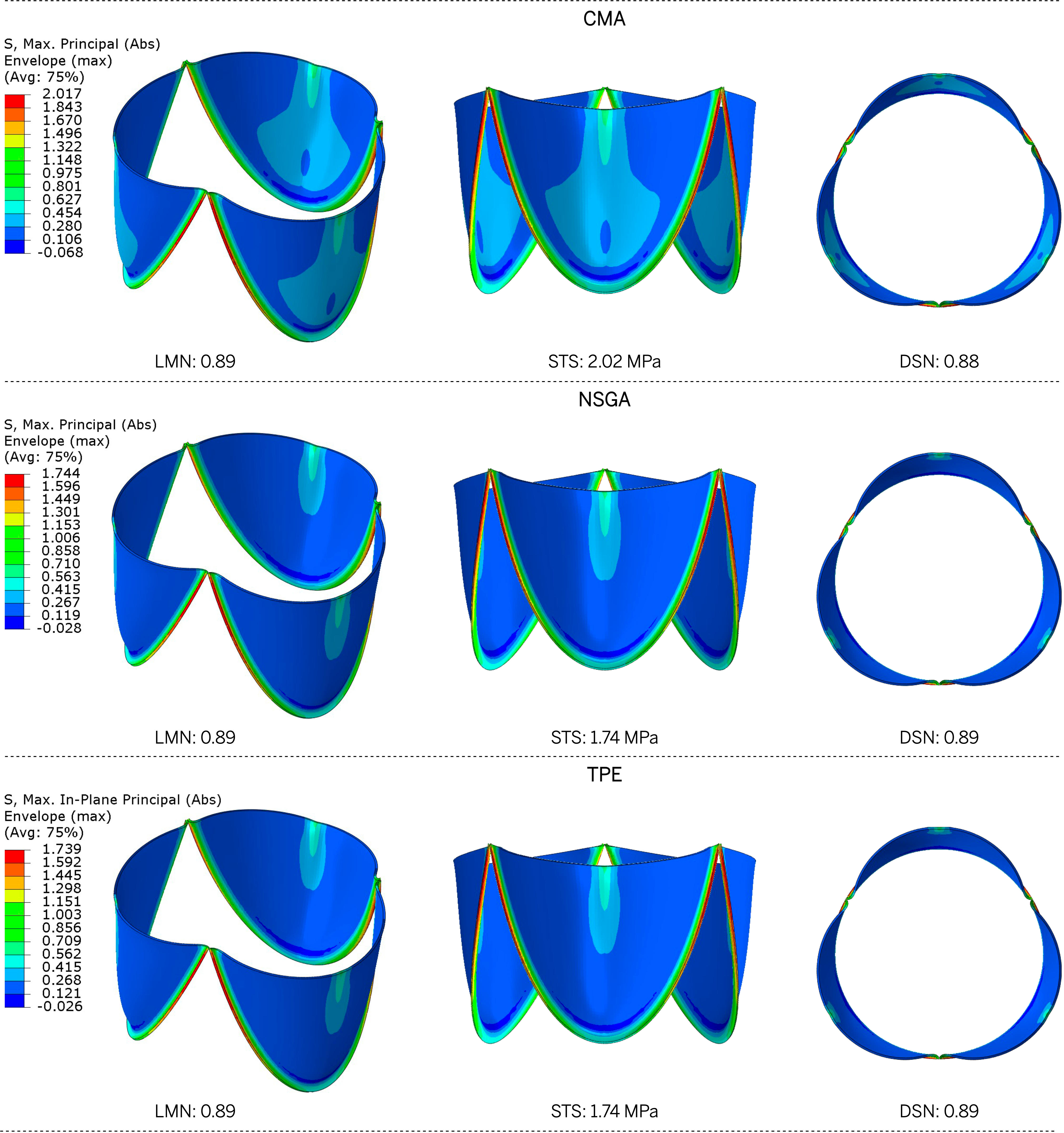

Supplement: Supplementary file 8 [file Image5.tif]
