# Supplementary material for: Perfect prosthetic heart valve: generative design with machine learning, modeling, and optimization
Source: Front Bioeng Biotechnol. 2023 Sep 15;11:1238130. doi: 10.3389/fbioe.2023.1238130 (PMC10541217; doi:10.3389/fbioe.2023.1238130)
Supplement: Supplementary file 12 [file DataSheet1.docx]

**Appendix A.** Mechanical properties of potential materials

| **#** | **Name** | **Type** | **ELM, MPa** | **UTS, MPa** | **Max deform, m/m** | **Reference** |
| --- | --- | --- | --- | --- | --- | --- |
| 1 | BioLab | Bovine xenopericardium | 19.4 | 12.72 | 67.42 | (Ovcharenko et al., 2016) |
| 2 | Bovine EGDE NeoCor | Bovine xenopericardium | 15.5 | 11.84 | 78.82 |  |
| 3 | Bovine GA RSCI | Bovine xenopericardium | 17.6 | 10.71 | 64.97 |  |
| 4 | Porcine GA RSCI | Porcine xenopericardium | 13.5 | 10.62 | 80.88 |  |
| 5 | Porcine EGDE RSCI | Porcine xenopericardium | 8.55 | 4.56 | 55.51 |  |
| 6 | Hastalex | Polymeric nanocomposite | 11.3 | 57.1 | 10.043 | (Ovcharenko et al., 2020) |
| 7 | Gore-tex (longitudinal) | ePTFE | 1.9 | 22.5 | 1.863 |  |
| 8 | Gore-tex (transverse) | ePTFE | 10.1 | 16.4 | 0.695 |  |
| 9 | SIBS160 | Polymer | 2.77 | 11.62 | 7.26 | (Rezvova et al., 2022) |
| 10 | SIBS160-CNT1 | Polymeric nanocomposite | 5.23 | 13.55 | 7.62 |  |
| 11 | SIBS160-CNT2 | Polymeric nanocomposite | 10.5 | 10.09 | 4.87 |  |
| 12 | SIBS160-CNT4 | Polymeric nanocomposite | 33.3 | 10.03 | 2.41 |  |
| 13 | SIBS129 | Polymer | 0.77 | 2.34 | 5.89 |  |
| 14 | SIBS129-CNT1 | Polymeric nanocomposite | 7.21 | 2.40 | 1.97 |  |
| 15 | SIBS129-CNT2 | Polymeric nanocomposite | 8.93 | 2.95 | 1.36 |  |
| 16 | SIBS129-CNT4 | Polymeric nanocomposite | 12.65 | 3.81 | 1.33 |  |
| 17 | Formlabs Elastic 50A | Resin  for 3D-printing | 2.02 | 3.23 | 1.6 | Manufacturer's datasheet ELM calculated at 160% |
| 18 | Formlabs Elastic 80A | Resin  for 3D-printing | 7.42 | 8.9 | 1.2 | Manufacturer's datasheet ELM calculated at 120% |
| 19 | Bovine GA (Hülsmann) | Bovine xenopericardium | 21 | 29 | 0.6 | (Aguiari et al., 2016) |
| 20 | Bovine GA (Bai) | Bovine xenopericardium | 10 | 6 | 0.5 |  |

**References**

Aguiari, P., Fiorese, M., Iop, L., Gerosa, G., and Bagno, A. (2016). Mechanical testing of pericardium for manufacturing prosthetic heart valves. *Interact Cardiovasc Thorac Surg* 22, 72–84. doi: 10.1093/icvts/ivv282.

Ovcharenko, E. A., Klyshnikov, K. Yu., Glushkova, T. V., Nyshtaev, D. V., Kudryavtseva, Y. A., and Savrasov, G. V. (2016). Xenopericardial Graft Selection for Valve Apparatus of Transcatheter Heart Valve Bioprosthesis. *Biomed Eng* 49, 253–257. doi: 10.1007/s10527-016-9543-0.

Ovcharenko, E. A., Seifalian, A., Rezvova, M. A., Klyshnikov, K. Y., Glushkova, T. V., Akenteva, T. N., et al. (2020). A New Nanocomposite Copolymer Based On Functionalised Graphene Oxide for Development of Heart Valves. *Scientific Reports* 2020 10:1 10, 1–14. doi: 10.1038/s41598-020-62122-8.

Rezvova, M. A., Nikishau, P. A., Makarevich, M. I., Glushkova, T. V., Klyshnikov, K. Y., Akentieva, T. N., et al. (2022). Biomaterials Based on Carbon Nanotube Nanocomposites of Poly(styrene-b-isobutylene-b-styrene): The Effect of Nanotube Content on the Mechanical Properties, Biocompatibility and Hemocompatibility. *Nanomaterials* 12, 733. doi: 10.3390/nano12050733.

**Appendix B.** Model performance during training and validation

*Table B1. Performance metrics for the studied models on both training and validation subsets*

| **№** | **Metric** | **LMN** | | | **STS** | | |
| --- | --- | --- | --- | --- | --- | --- | --- |
|  |  | **Train** | **Validation** | **Test** | **Train** | **Validation** | **Test** |
| 1 | MAPE | 0.116 | 0.118 | 0.163 | 0.093 | 0.102 | 0.086 |
| 2 | WAPE | 0.042 | 0.044 | 0.032 | 0.077 | 0.081 | 0.074 |
| 3 | MAE | 0.018 | 0.019 | 0.018 | 0.127 | 0.135 | 0.075 |
| 4 | MAAPE | 0.096 | 0.099 | 0.089 | 0.090 | 0.098 | 0.084 |
| 5 | MASE | 0.048 | 0.050 | 0.087 | 0.073 | 0.076 | 0.118 |
| 6 | MSE | 0.002 | 0.002 | 0.002 | 0.080 | 0.087 | 0.031 |
| 7 | RMSE | 0.040 | 0.043 | 0.044 | 0.284 | 0.294 | 0.177 |
| 8 | NRMSE | 0.040 | 0.044 | 0.041 | 0.022 | 0.029 | 0.021 |
| 9 | R^2^ | 98.7% | 98.4% | 98.6% | 97.0% | 96.8% | 95.5% |
| 10 | PCC | 99.3% | 99.2% | 99.3% | 98.5% | 98.4% | 97.9% |
| 11 | ME | -0.001 | -0.002 | -0.003 | 0.014 | 0.015 | 0.039 |
| 12 | MAD | 0.018 | 0.019 | 0.018 | 0.127 | 0.135 | 0.075 |
| 13 | GMAE | 0.007 | 0.007 | 0.007 | 0.038 | 0.040 | 0.033 |
| 14 | MDAE | 0.007 | 0.008 | 0.009 | 0.040 | 0.046 | 0.040 |
| 15 | MPE | -0.036 | -0.044 | -0.069 | -0.029 | -0.032 | 0.019 |
| 16 | MDAPE | 0.035 | 0.036 | 0.018 | 0.054 | 0.059 | 0.055 |
| 17 | SMAPE | 0.102 | 0.105 | 0.114 | 0.089 | 0.098 | 0.087 |
| 18 | SMDAPE | 0.035 | 0.036 | 0.018 | 0.054 | 0.058 | 0.055 |
| 19 | STDAE | 0.044 | 0.048 | 0.049 | 0.305 | 0.317 | 0.176 |
| 20 | RMDSPE | 0.035 | 0.036 | 0.018 | 0.054 | 0.059 | 0.055 |
| 21 | RMSSE | 0.105 | 0.115 | 0.209 | 0.162 | 0.167 | 0.279 |
| 22 | INRSE | 0.116 | 0.128 | 0.119 | 0.173 | 0.179 | 0.213 |
| 23 | RRSE | 0.116 | 0.128 | 0.119 | 0.173 | 0.179 | 0.213 |
| 24 | RAE | 0.056 | 0.059 | 0.054 | 0.094 | 0.100 | 0.128 |
| 25 | MDA | 0.967 | 0.971 | 0.897 | 0.972 | 0.968 | 0.886 |

*
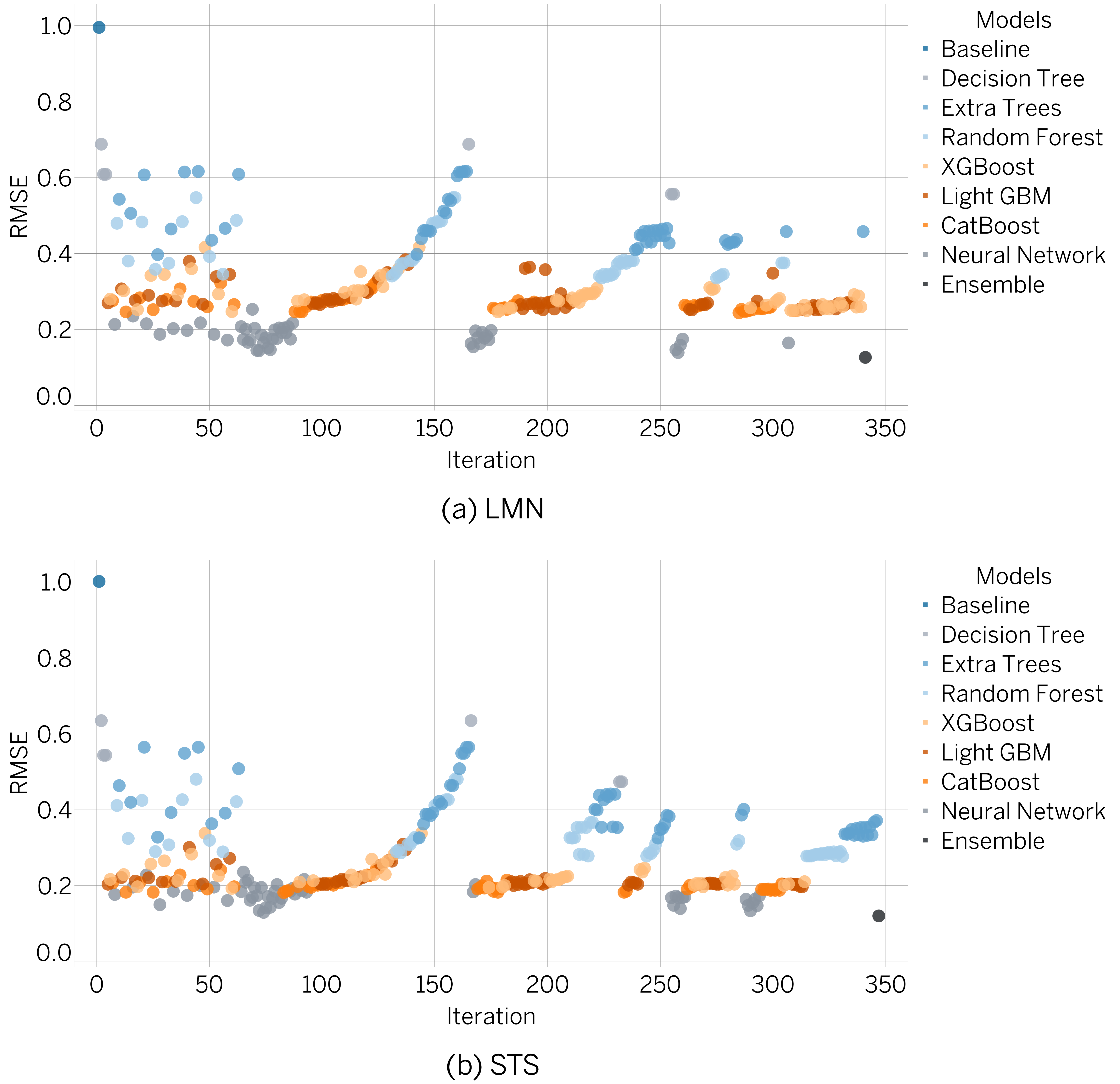
*

*Figure B1. Distribution of RMSE for studied models during training*

**Appendix C.** Shapley values for the PHV parameters under study


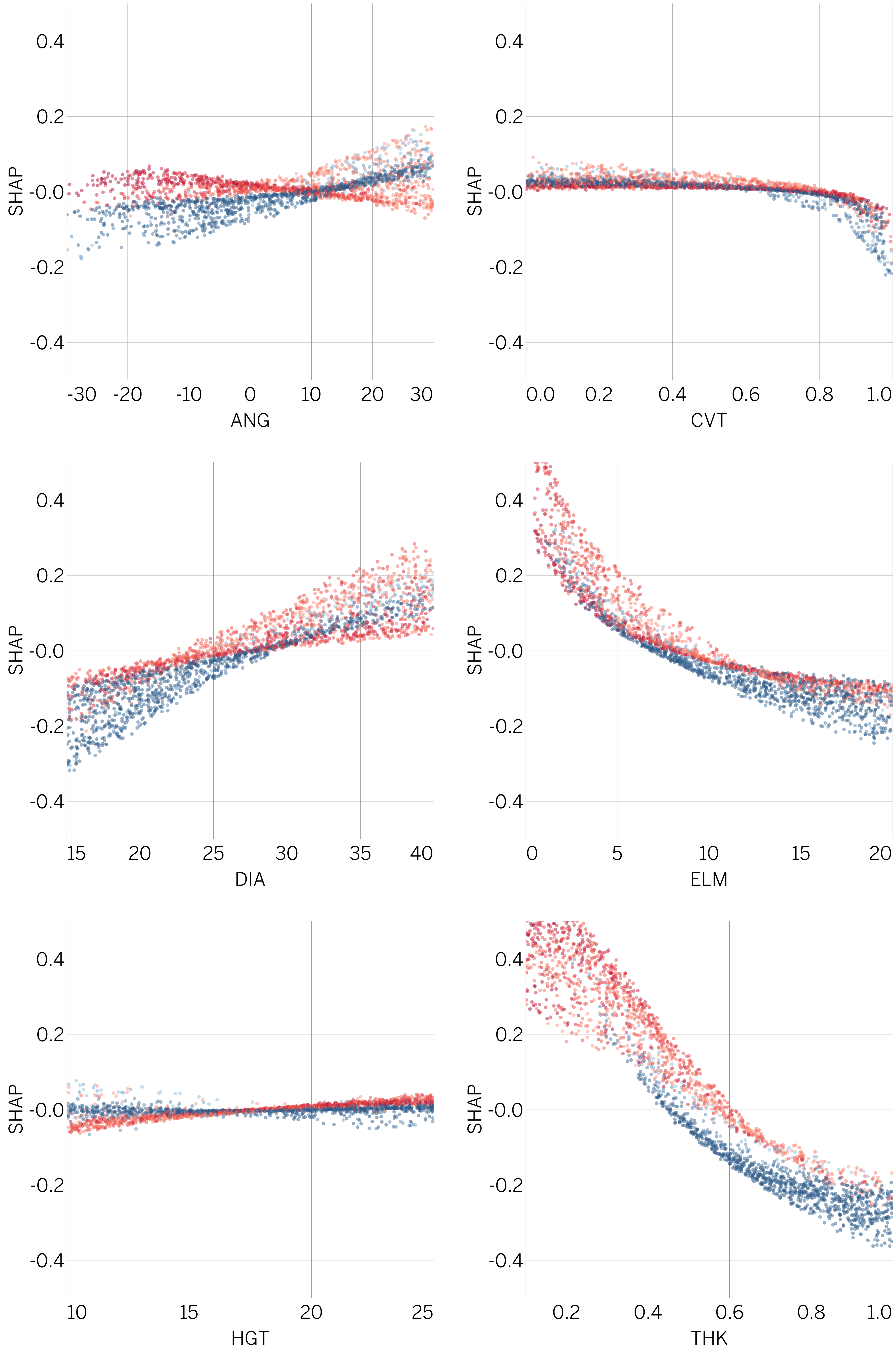


*Figure C1. Shapley values for the model predicting lumen value (LMN). Blue and red dots represent lower and higher lumen values of a given PHV design*

*
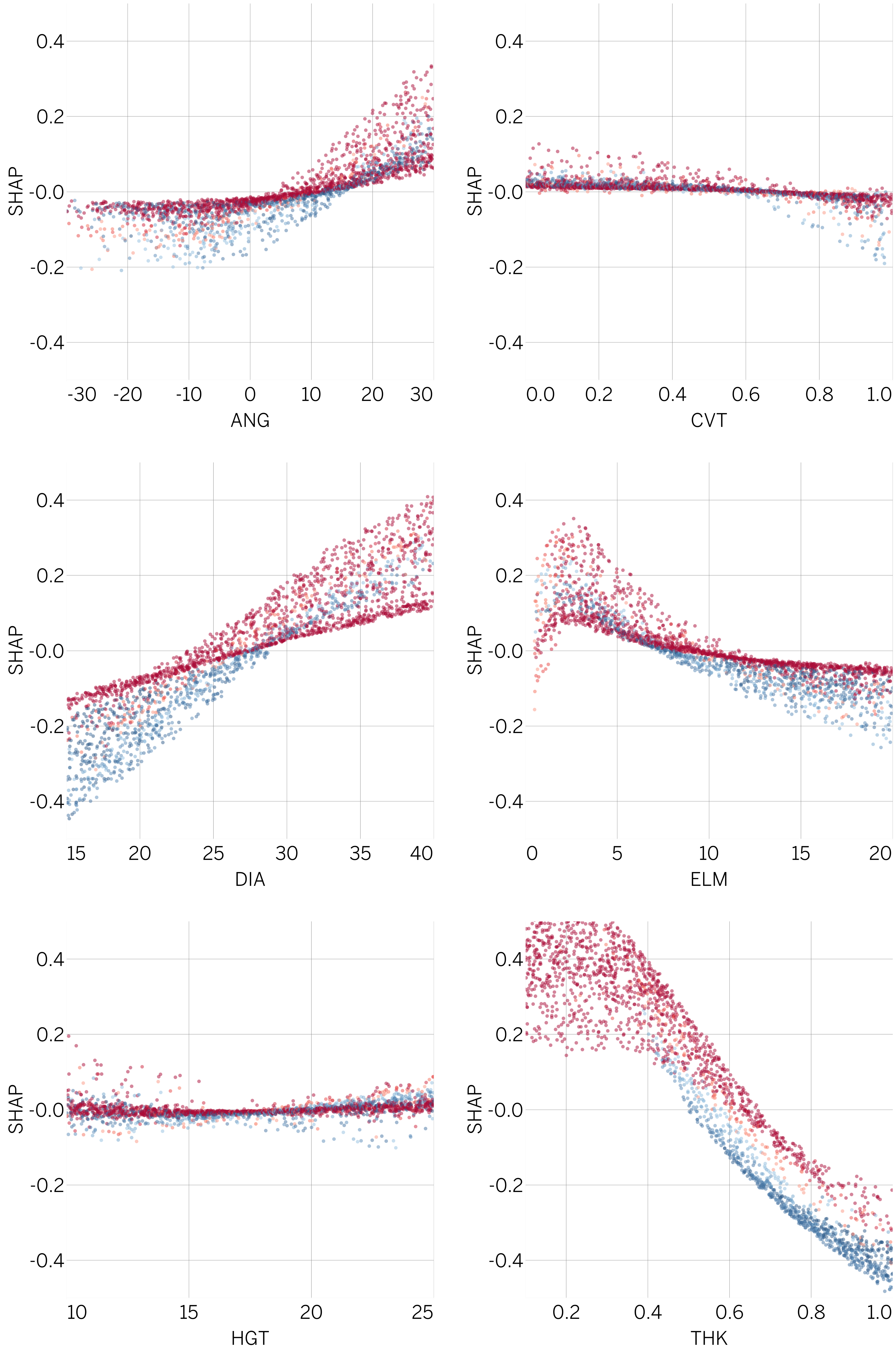
*

*Figure C2. Shapley values for the model predicting stress value (STS). Blue and red dots represent lower and higher stress values of a given PHV design*

**Appendix D.** Leaflet designs featuring minimized principal stress and optimal leaflet opening


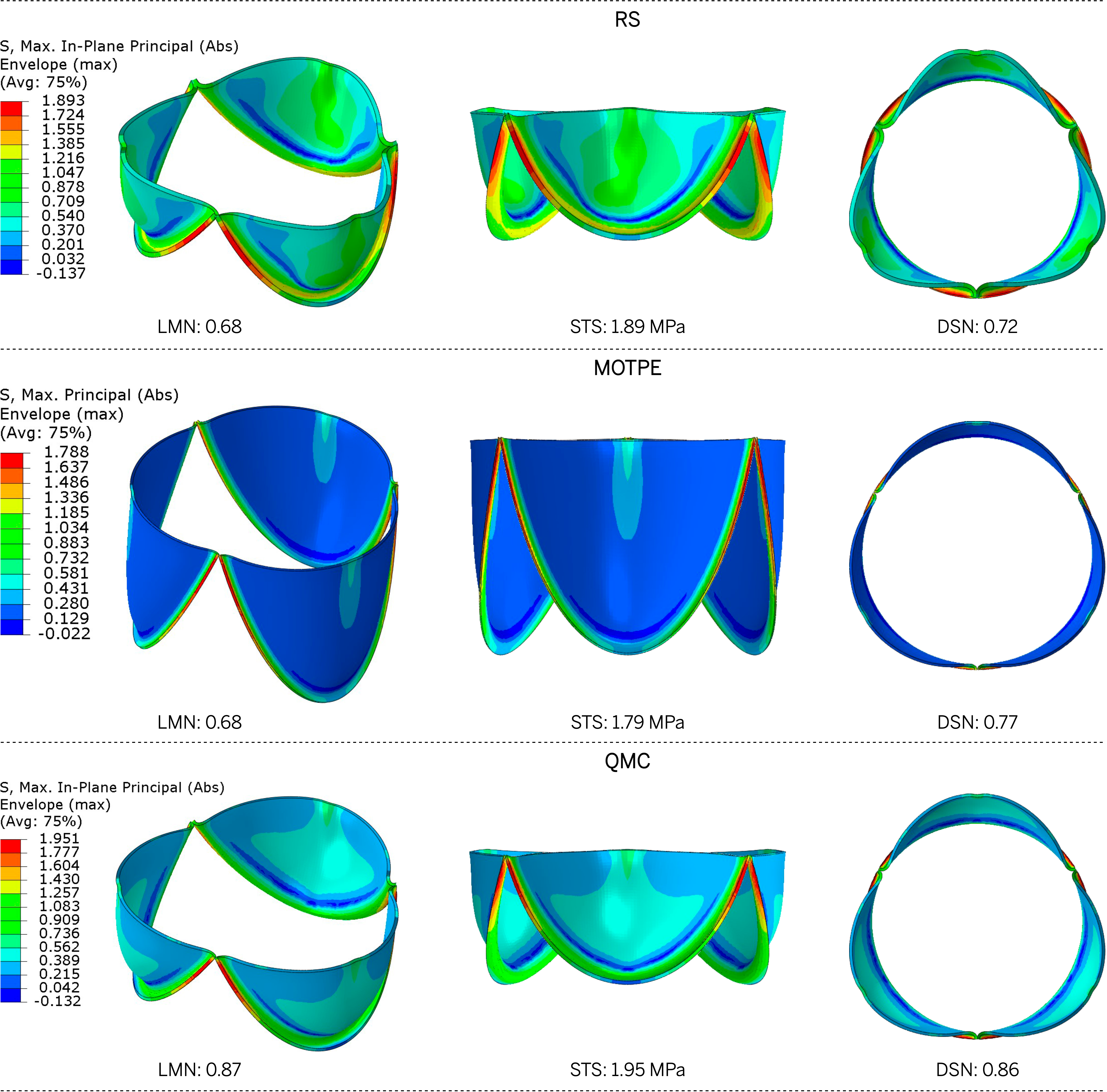


**
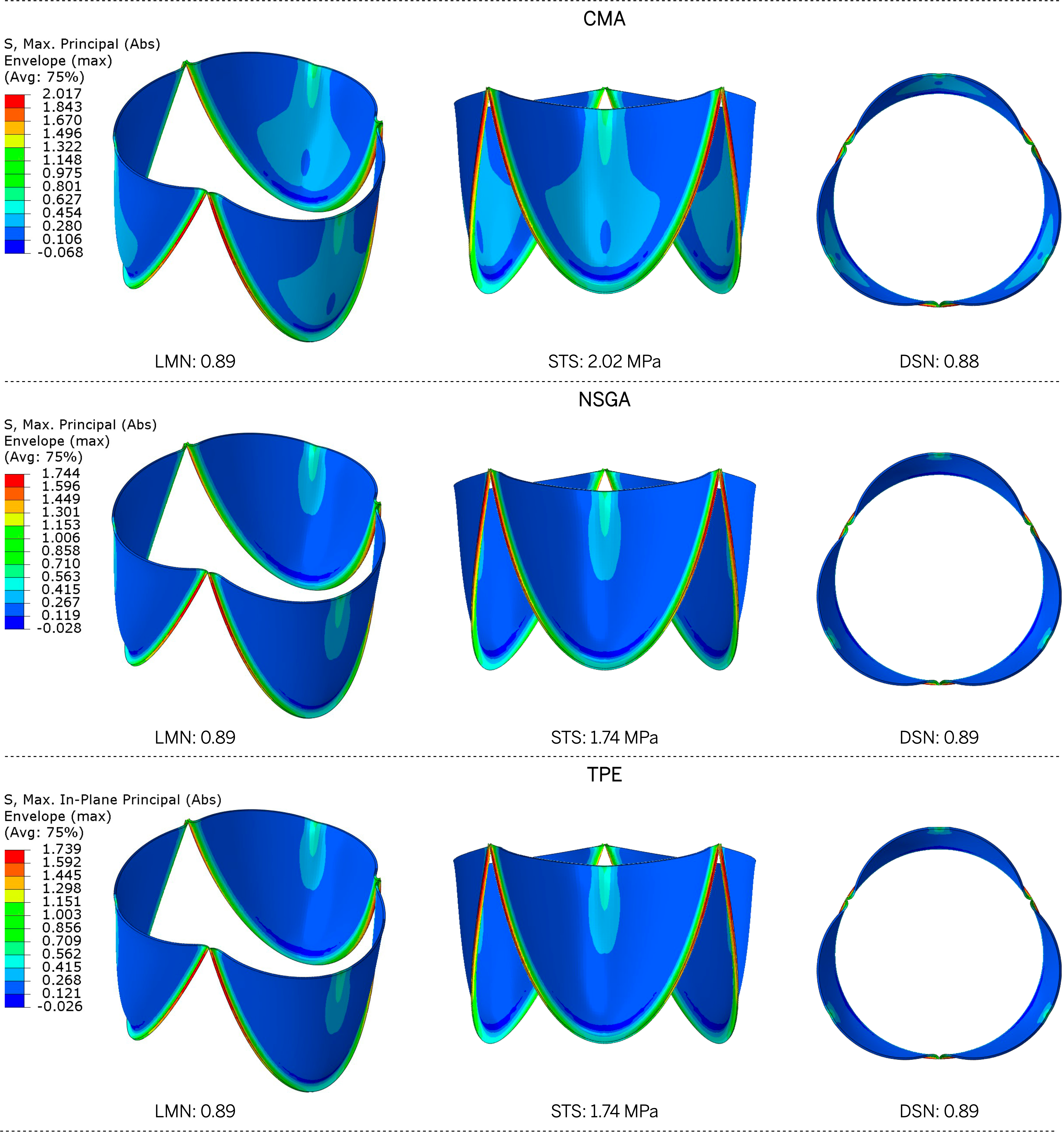
**
